# Single-cell profiling of alveolar rhabdomyosarcoma reveals RAS pathway inhibitors as cell-fate hijackers with therapeutic relevance

**DOI:** 10.1126/sciadv.ade9238

**Published:** 2023-02-08

**Authors:** Sara G. Danielli, Ermelinda Porpiglia, Andrea J. De Micheli, Natalia Navarro, Michael J. Zellinger, Ingrid Bechtold, Samanta Kisele, Larissa Volken, Quy A. Ngo, Joana G. Marques, Stephanie Kasper, Peter K. Bode, Anton G. Henssen, Dennis Gürgen, Olivier Delattre, Didier Surdez, Josep Roma, Peter Bühlmann, Helen M. Blau, Marco Wachtel, Beat W. Schäfer

**Affiliations:** ^1^ Department of Oncology and Children’s Research Center, University Children’s Hospital of Zurich, Zürich 8032, Switzerland.; ^2^ Baxter Laboratory for Stem Cell Biology, Department of Microbiology and Immunology, Institute for Stem Cell Biology and Regenerative Medicine, Stanford University School of Medicine, Stanford, CA 94305, USA.; ^3^ Department of Biomedicine, Aarhus University, Aarhus C 8000, Denmark.; ^4^ Laboratory of Translational Research in Child and Adolescent Cancer, Vall d’Hebron Research Institute, Hospital Universitari Vall d’Hebron, Universitat Autònoma de Barcelona, Barcelona 08035, Spain.; ^5^ Seminar for Statistics, ETH Zürich, Zürich 8092, Switzerland.; ^6^ Department of Pathology, University Hospital Zurich, Zurich, Switzerland.; ^7^ Department of Pediatric Oncology/Hematology, Charité–Universitätsmedizin Berlin, Berlin 13353, Germany.; ^8^ EPO Experimental Pharmacology and Oncology Berlin-Buch GmbH Berlin 13125, Germany.; ^9^ INSERM U830, Équipe Labellisée LNCC, Diversity and Plasticity of Childhood Tumors Laboratory, PSL Research University, SIREDO Oncology Center, Institut Curie Research Center, Paris 75005, France.; ^10^ Balgrist University Hospital, Faculty of Medicine, University of Zurich (UZH), Zurich, Switzerland.

## Abstract

Rhabdomyosarcoma (RMS) is a group of pediatric cancers with features of developing skeletal muscle. The cellular hierarchy and mechanisms leading to developmental arrest remain elusive. Here, we combined single-cell RNA sequencing, mass cytometry, and high-content imaging to resolve intratumoral heterogeneity of patient-derived primary RMS cultures. We show that the aggressive alveolar RMS (aRMS) subtype contains plastic muscle stem-like cells and cycling progenitors that drive tumor growth, and a subpopulation of differentiated cells that lost its proliferative potential and correlates with better outcomes. While chemotherapy eliminates cycling progenitors, it enriches aRMS for muscle stem-like cells. We screened for drugs hijacking aRMS toward clinically favorable subpopulations and identified a combination of RAF and MEK inhibitors that potently induces myogenic differentiation and inhibits tumor growth. Overall, our work provides insights into the developmental states underlying aRMS aggressiveness, chemoresistance, and progression and identifies the RAS pathway as a promising therapeutic target.

## Teaser

RMS contain subpopulations recapitulating muscle developmental stages, which can be hijacked towards differentiation.

## INTRODUCTION

Childhood cancer is a disease of dysregulated human development (*1*, *2*) and the leading cause of disease-related morbidity and mortality in childhood (*3*). Recent technical advances in single-cell technologies have helped in characterizing intratumoral heterogeneity and phenotypic plasticity in several cancer types, highlighting their role as emerging hallmarks of cancer (*4*). Single-cell RNA-sequencing (scRNAseq) studies of pediatric solid tumors, including glioma, neuroblastoma, ependymoma, and medulloblastoma, demonstrated that these cancers contain subpopulations of cells with different degrees of differentiation that mirror the developmental stages of the putative tissues of origin (*5*–*8*). Therefore, characterizing the developmental hierarchies of childhood tumors could be instrumental in identifying the cell of origin and for developing effective treatments that target the relevant cellular compartments.

Rhabdomyosarcoma (RMS) is the most common pediatric soft tissue sarcoma (*9*). The two main RMS subtypes include embryonal RMS (eRMS) and alveolar RMS (aRMS), the more aggressive form, driven by a chromosomal translocation between *PAX3* or *PAX7* and the *FOXO1* genes (*9*). RMS tumors are believed to originate from differentiation defects during skeletal muscle development (*10*). Despite expression of the transcription factors that drive myogenic commitment and differentiation, such as myoblast determination protein 1 (*MYOD1*) and myogenin (*MYOG*) (*11*, *12*), differentiation is blocked in RMS cells, which therefore resemble muscle progenitor cells. Previous reports proposed different stages along the myogenic lineage as the candidate cells of origin for RMS, including mesenchymal progenitors (*13*), satellite cells (*14*), and/or mature differentiated myofibers (*15*). More recent work implicated nonmyogenic cells of origin, such as endothelial progenitor cells (*16*, *17*). However, the exact RMS cell of origin remains a matter of debate. Resolving the developmental heterogeneity and the mechanisms that lead to developmental arrest in RMS tumors is necessary to identify the cell origin and inform therapeutic strategies.

One-third of localized and two-third of metastatic RMS cases exhibit relapse or disease progression despite intense treatments (*18*, *19*). In such cases, prognosis is dismal and 5-year overall survival rates drop below 10% for the aRMS subtype (*19*). While some eRMS tumors harbor druggable genetic alterations, the mutational landscape of aRMS tumors consists mainly of the oncogenic *PAX3/7::FOXO1* fusion itself (*20*, *21*). PAX3::FOXO1 is a potent and aberrant chimeric transcription factor that regulates proliferation, survival, and differentiation of aRMS cells, therefore representing an ideal but currently undruggable therapeutic target (*22*). This highlights the urgent need to identify therapeutic approaches in relapsed aRMS settings.

Differentiation therapy has been long entertained as a promising therapeutic option in childhood cancers (*1*, *23*, *24*). Despite the promises of a treatment that would restore terminal maturation of tumor cells, with consequent loss of their malignant phenotype, clinical applications are still rare. Recent studies demonstrated that myogenic differentiation can be triggered in *RAS*-mutant eRMS cells by interfering with RAS signaling (*25*–*27*), while therapeutic differentiation of the more aggressive aRMS subtype has not been reported to date. A better understanding of the differentiation pathways in aRMS is essential for designing effective differentiation therapies.

Here, we used a combination of scRNAseq, mass cytometry [cytometry by time of flight (CyTOF)], and high-content imaging analysis to characterize intratumoral heterogeneity of RMS cell lines and primary cultures derived from patient-derived xenografts (PDXs). We uncovered a variety of subpopulations mapping to developing or regenerating skeletal muscle cells and show that the immature muscle stem-like (MuSC-like) subpopulation faces a bifurcated cell-fate decision. The identified signatures revealed to be strongly associated with aRMS patient survival, with the differentiated signature being enriched in patients with a good clinical course. We therefore screened for regulators of aRMS cellular fate using a library of pharmacological compounds and identified combinations of RAS pathway inhibitors, trametinib with dabrafenib or regorafenib, which direct aRMS cells toward differentiation. We further show that these drug combinations potently suppress PDX tumor growth, providing a strong rationale for the clinical manipulation of the RAS pathway in aRMS patients and for inducing differentiation to counter oncogenicity.

## RESULTS

### scRNAseq identifies distinct muscle developmental states in RMS

**Fig. 1.**
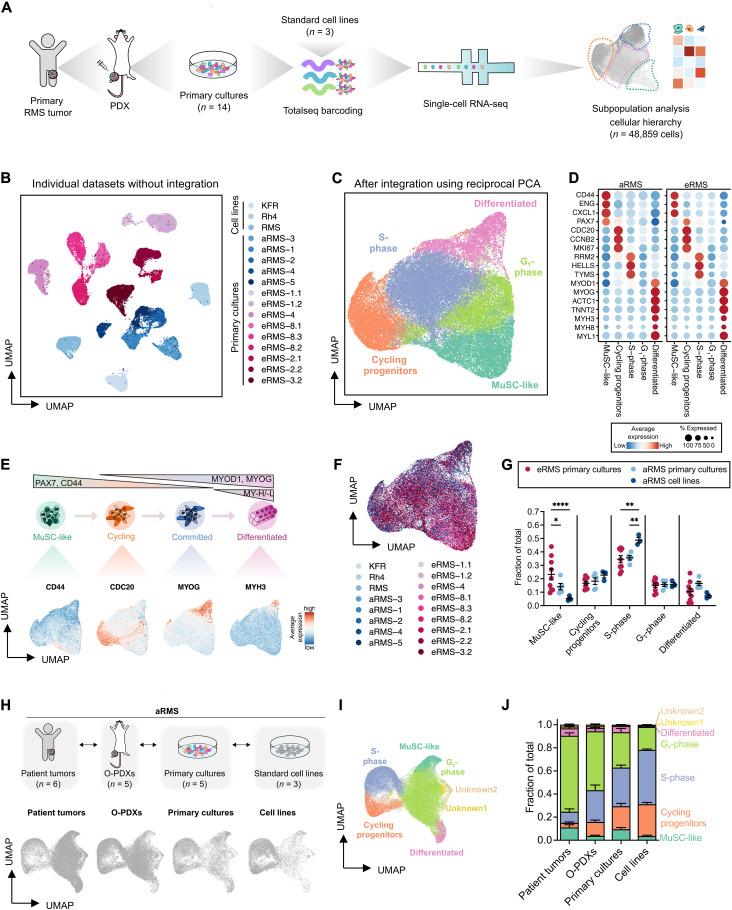
scRNAseq of RMS identifies heterogeneity recapitulating muscle developmental programs. (**A**) Experimental workflow. (**B**) UMAP plot of 48,859 RMS cells after regressing the number of count RNA, the percentage of mitochondrial genes, and the run batch effect. Cells are color-coded based on the corresponding sample of origin. (**C**) UMAP plot of 48,859 RMS cells after integration. Populations identified by Louvain clustering are shown. (**D**) Dot plot showing expression of lineage-specific marker genes across the different Louvain clusters in aRMS and eRMS samples. (**E**) Model of skeletal myogenesis with the populations identified in RMS. UMAP plots are colored on the basis of the expression of markers delineating a myogenic lineage progression. (**F**) UMAP plot of RMS cells after integration and color-coded based on the sample of origin. (**G**) Relative proportion of Louvain clusters. Data are represented as means ± SEM; ordinary two-way analysis of variance (ANOVA) with uncorrected Fisher’s least significant difference (LSD). *P < 0.05; **P < 0.01; ****P ≤ 0.0001. (**H** and **I**) Comparison of intratumoral heterogeneity between preclinical models of aRMS. O-PDXs and patient tumor data are derived from (*36*). UMAP plots of individual models (H) and of the integrated datasets (I) are shown. (**J**) Relative proportion of Louvain clusters across different aRMS preclinical models and patient tumors. Data are represented as means ± SEM of *n* = 6 patient tumors, *n* = 5 O-PDXs, *n* = 5 primary cultures, and *n* = 3 cell lines.

To characterize intratumoral heterogeneity of RMS, we profiled 14 RMS PDX-derived primary cultures and 3 conventional aRMS cell lines by droplet-based scRNAseq (Fig. 1A). To maximize interpatient variability, we chose RMS models that originate from both aRMS and eRMS subtypes, primary and metastatic sites, and diagnostic and recurrent patients that had or had not been pretreated (table S1). After filtering out low-quality cells, we retained 48,859 cells for downstream analysis. Cells clustered mainly by patient of origin (Fig. 1B), indicating substantial intertumor heterogeneity, a characteristic previously described for other tumor types (*28*). To identify shared cell subpopulations and gene expression signatures, we integrated the RMS datasets using the “anchor” approach (*29*). Unsupervised clustering resolved five distinct groups of cells that shared similar transcriptomic signatures (Fig. 1C, fig. S1A, and table S2). We used gene set enrichment analysis (*30*) in addition to published marker genes (table S3) (*31*, *32*), to group the cells into transcriptionally distinct cellular states. These cellular states included an early myogenic subpopulation (MuSC-like), which resembled muscle stem cells (MuSCs) and was enriched in vasculature development and cell adhesion genes, a proliferating subpopulation (cycling progenitor) that expressed high levels of cell division and cell cycle genes, and a subpopulation of differentiated cells (differentiated), enriched in muscle system processes and myotube differentiation genes (fig. S1, B and C). We observed the same cellular states when integrating only aRMS or eRMS primary cultures or cell lines (fig. S1D and table S2). In addition, we identified a subpopulation of cells in the G_1_ cell cycle phase that failed to express specific gene signatures (G_1_-phase), and another that was enriched in DNA replication processes and that expressed genes characteristic of cells in the S phase (S-phase) (fig. S1, A and B) (*33*). These cells expressed intermediate levels of *MYOD1* (Fig. 1D) and high levels of *MYOG* in aRMS (fig. S1E) or of *PAX7* in eRMS (Fig. 1D), indicative of transitory cells located between MuSC-like and differentiated subpopulations in the myogenic lineage. The mesenchymal/muscle progenitor markers *CD44*, *ENG* (which encodes for CD105), *CXCL1*, and *PAX7* were mainly expressed by MuSC-like cells; the proliferation markers *CDC20*, *CCNB1*, and *MKI67* were expressed by cycling progenitors; the committed muscle markers *MYOG*, *ACTC1*, and *TNNT2* exhibited increased expression in cells undergoing differentiation; and expression of myosin heavy (MyHCs) and light chain (e.g. *MYH3*, *MYH8*, and *MYL1*) was exclusive of the differentiated subpopulation (Fig. 1, D and E). MuSC-like cells were the only aRMS subpopulation clearly characterized by the absence of *MYOG* and expression of *CD44*, *PAX7*, and *AXL* (fig. S1E). To distinguish high-cycling from low-cycling cells, we scored each cell for validated gene signatures (*34*). Notably, most MuSC-like and differentiated cells were low cycling, whereas cells in S-phase and cycling progenitors were high cycling (fig. S2A). We noted that eRMS mapped mostly to the MuSC-like cluster, whereas aRMS cell lines contained a higher proportion of cells in the cycling S-phase cluster compared to eRMS or primary cultures (Fig. 1, F and G). We confirmed these differences in a large RMS patient mRNA expression dataset (*35*). Compared to eRMS, aRMS patients showed significantly lower *PAX7*, known to mark MuSCs, higher *MYOD1* and *MYOG*, known to mark activated and committed cells, and no difference in *MYH8* mRNA expression, known to mark differentiated myogenic cells (fig. S2B).

We wondered whether primary cultures and cell lines maintain the heterogeneity of the originating tumors, and therefore expanded our single-cell transcriptomic profiling to include orthotopic PDXs (O-PDXs) and patient tumors recently profiled by Patel *et al.* (*36*). We first focused on three eRMS samples, which were sequenced in both studies, and observed that primary cultures shared similar transcriptomic spaces of the originating O-PDXs and, when available, of the patient tumor (fig. S2C). As there were no matched aRMS samples, we compared the heterogeneity of aRMS cell lines and primary cultures profiled in our study with O-PDXs and patient tumors profiled by Patel *et al.* (*36*). Patient tumors mainly mapped onto MuSC-like, G_1_-phase, and differentiated clusters and had fewer cells in the proliferating S-phase and cycling progenitor clusters, which were instead overrepresented in cell lines; primary cultures maintained a similar frequency of MuSC-like and differentiated cells than the patient tumors but higher abundance of S-phase and lower abundance of G_1_-phase cells (Fig. 1, H to J, and fig. S2D). Together, these findings support the notion that RMS tumors contain myogenic cells stalled in an immature transcriptional state, but they reveal a cellular continuum that spans the entire myogenic lineage from MuSC-like to a minority of differentiated cells.

### CyTOF reveals cell surface markers associated with the immature MuSC-like aRMS subpopulation

**Fig. 2.**
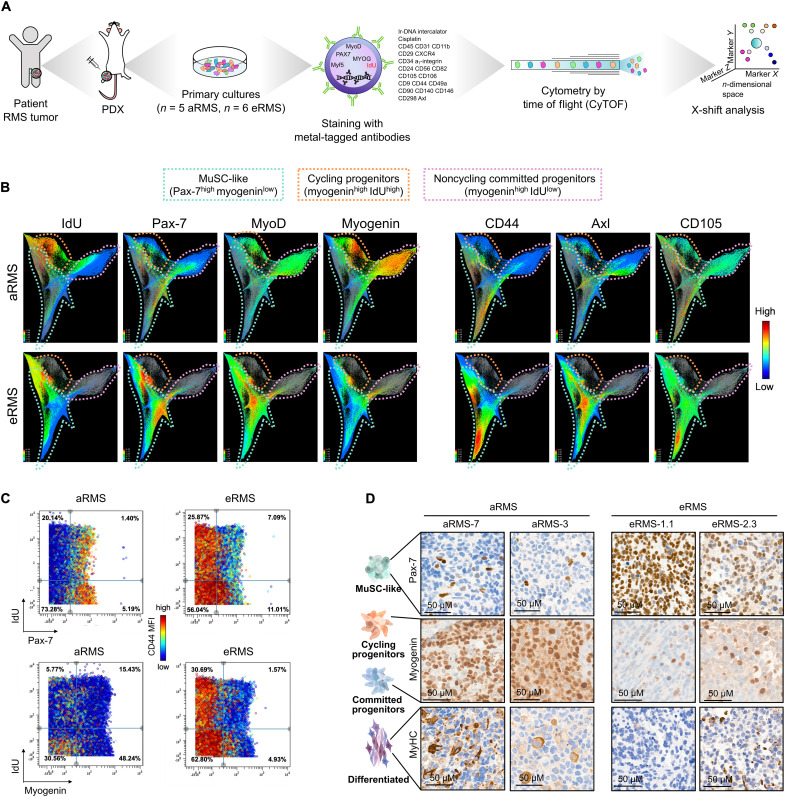
CyTOF defines RMS subpopulations with high resolution. (**A**) Mass cytometry workflow. (**B**) Integrated CyTOF dataset from RMS primary cultures clustered with the X-shift algorithm and visualized using single-cell force-directed layout. (**C**) Biaxial dot plots of Pax-7 or myogenin by IdU across aRMS and eRMS primary cultures. Plots are colored by CD44 expression. (**D**) Expression of Pax-7, myogenin, and MyHC markers as determined by immunohistochemistry in PDX tumors.

To confirm the presence of the identified subpopulations at the protein level, we stained primary RMS cultures with an isotope-conjugated antibody panel, which included known surface markers used to isolate MuSCs as well as identified by scRNAseq, and myogenic transcription factors known to define distinct stages of myogenesis (*37*), and profiled the cells by CyTOF. We analyzed the pooled aRMS and eRMS datasets with the clustering algorithm X-shift (*38*) and visualized the spatial relationship between the resulting clusters by single-cell force-directed layout (Fig. 2A). While cells from aRMS and eRMS shared some commonalities, they also occupied unique regions that were specific to each subtype (Fig. 2B). We defined cells that were actively incorporating 5′-iododeoxyuridine (IdU), a marker of S-phase, as high-cycling cells, whereas cells that were not incorporating IdU were defined as low-cycling cells (*39*). Pax-7^high^ cells were present in both subsets, which led to identification of quiescent MuSC-like and proliferating MuSC-like cells in both aRMS and eRMS samples (Fig. 2. C). We found that, in aRMS, the cell surface proteins CD44, Axl, and CD105 marked the Pax-7^high^/myogenin^low^ MuSC-like cells (Fig. 2, B and C), providing a strategy to prospectively isolate this subpopulation for in-depth functional studies. A unique signature of aRMS includes the presence of two prominent myogenin^high^ subsets (Fig. 2, B and C), one of which incorporated high levels of IdU and is therefore cycling (cycling progenitors). This subset is of particular interest because it proliferates and, at the same time, expresses the differentiation marker myogenin, which, in muscle stem and progenitor cells, causes differentiation and cell cycle exit.

We also noted that Pax-7 expression was higher in eRMS cells and myogenin expression was higher in aRMS cells (Fig. 2C). These findings are in line with the scRNAseq analysis described above, suggesting that eRMS maps onto immature muscle stages, whereas aRMS represents more mature stages. To further confirm the differential cellular distribution of aRMS and eRMS, we quantified the proportion of cells arrested at progenitor (Pax-7^+^), activated (MyoD^+^), committed (myogenin^+^), and differentiated stages (MyHC^+^) by CyTOF, immunofluorescence, WB, and immunohistochemical staining of PDX tumors. Again, we uncovered heterogeneity in the expression of myogenic markers, with aRMS mapping to more mature muscle stages than eRMS tumors (Fig. 2D and fig. S2, E to G).

In summary, our single-cell protein analysis corroborates the transcriptomic analysis. We identified four developmental RMS stages, which include quiescent MuSC-like cells (IdU^low^/Pax-7^high^/myogenin^low^), cycling MuSC-like cells (IdU^high^/Pax-7^high^/myogenin^low^), cycling progenitors defective in differentiation (IdU^high^/myogenin^high^), and noncycling committed/differentiated progenitors (IdU^low^/myogenin^high^).

### RMS mirror cell-fate decisions of developing/regenerating skeletal MuSCs

**Fig. 3.**
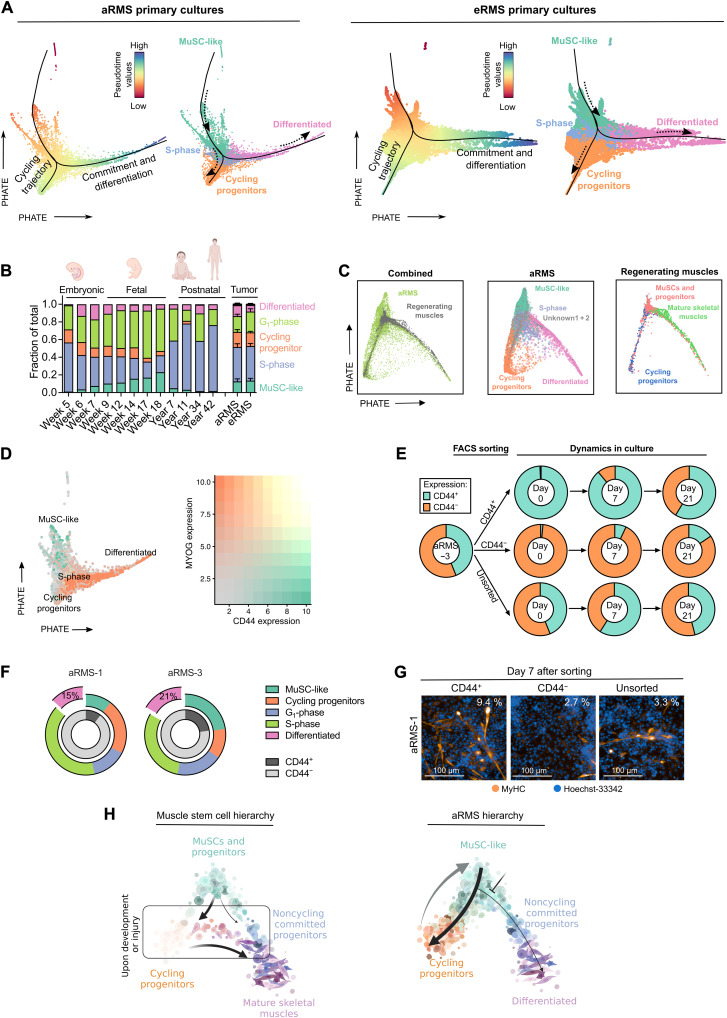
RMS primary cultures recapitulate a branched myogenic trajectory. (**A**) PHATE dimensionality reduction (*t* = 30, *knn* = 20) plots of aRMS (left) or eRMS (right) primary cultures. Black lines represent pseudotime trajectories calculated using Slingshot (starting cluster: MuSC-like); dashed arrows represent the trajectory direction. Cells are colored on the basis of pseudotime values calculated by Slingshot (left) or on the identified Louvain clusters (right). (**B**) Clustering distribution of the integrated RMS/human developing skeletal muscle (*42*) dataset across developmental time points or RMS subtype. (**C**) PHATE dimensionality reduction (*t* = 30, *knn* = 20) plot of the integrated aRMS primary culture/mouse regenerating skeletal muscle (*32*) dataset. aRMS cells are color-coded on the basis of Louvain clusters; muscle cells are colored on the basis of the clusters identified in the original publication. (**D**) PHATE dimensionality reduction (*t* = 30, *knn* = 20) plot of aRMS primary cultures colored on the basis of *CD44* (green) or *MYOG* (orange) expression (left plot). The two markers are mutually exclusive (right plot). (**E**) Flow cytometry analysis of sorted CD44^+^ and CD44^−^ subpopulations in aRMS-3 cells. Unsorted reference is also shown. Data are represented as means of *n* ≥ 2 biological replicates. (**F**) Relative proportion of Louvain clusters across aRMS-1 and aRMS-3 cells before sorting. The percentage of differentiated cells in the CD44^−^ subpopulation is shown. (**G**) Immunofluorescence analysis of MyHC expression 7 days after sorting of CD44^+^ and CD44^−^ subpopulations in aRMS-1 cells. The percentage of MyHC^+^ cells is indicated on the top right of each panel. (**H**) Proposed model of aRMS hierarchical structure compared to developing or regenerating MuSCs.

We investigated the relationship between different RMS subpopulations using a trajectory inference model. For each dataset, we inferred trajectories and pseudotime values using Slingshot (*40*), and embedded the single cells using PHATE (*41*). We excluded two clusters (“unknown 1” and “unknown 2”) from aRMS primary cultures, as these subpopulations were present only in two specific samples, which might be the result of incomplete batch effect removal (fig. S3A). In both aRMS and eRMS, the predicted trajectory revealed a branched progression from MuSC-like cells to either cycling progenitors or differentiated cells (Fig. 3A).

To identify developmental programs that are aberrantly persistent in RMS, we reanalyzed an scRNAseq atlas of developing human skeletal muscles (*42*). Projection of our RMS single-cell transcriptomes onto this dataset (fig. S3B) showed that tumor cells mostly map onto muscle cells transitioning from embryonic to fetal stages (weeks 6 to 14; Fig. 3B). Similarly, we projected our primary aRMS cultures onto an scRNAseq dataset of regenerating skeletal muscles (*32*) and observed overlap of the branched cell-fate trajectories (Fig. 3C). Notably, the cycling progenitor subpopulation, which is only transiently detected in skeletal muscle cells upon injury (fig. S3C), represents a prominent and stable subpopulation in aRMS tumors.

To validate the predicted cellular trajectory of aRMS tumors, we took advantage of the cell surface marker CD44, which we found to be predominantly expressed on MuSC-like cells (Figs. 2, B and C, 3D and fig. S1E). We first measured CD44 expression across RMS primary cultures and cell lines and then focused our study on aRMS-1 (IC-pPDX-104) and aRMS-3 (IC-pPDX-35), as they exhibited two distinct CD44^+^ and CD44^−^ subpopulations (fig. S3D). We assessed the feasibility of using CD44 as a marker of MuSC-like cells by measuring the transcript levels of key myogenic markers in CD44^+^ and CD44^−^ cell subpopulations that were purified by fluorescence-activated cell sorting (FACS). CD44^+^ cells exhibited increased expression of *CD44* and *PAX7*, and decreased expression of *MYOD1* and *MYOG*, compared to CD44^−^ cells (fig. S3E), supporting the rationale of using CD44 as a MuSC-like marker. We sought to determine whether the trajectory of aRMS primary cultures is unidirectional or whether aRMS cells dedifferentiate to a MuSC-like state. We therefore separated MuSC-like CD44^+^ cells from the other subpopulations (CD44^−^), which mainly encompass cycling progenitors, S-phase, and a small percentage of differentiated cells, and monitored their behavior in culture (fig. S3F). We quantified CD44 expression in the sorted cells over time using flow cytometry and found that both CD44^+^ and CD44^−^ subpopulations regained their initial mixed composition after 2 weeks in culture (Fig. 3E and fig. S3G).

Because CD44^+^ cells lost CD44 expression faster than CD44^−^ gained it, we wondered whether the presence of CD44^+^ cells in the CD44^−^ sorted subpopulation was due to self-renewal of some remaining MuSC-like cells that were not completely removed during sorting. However, we observed similar proliferation (fig. S3H) and cell cycle distributions of both subpopulations (fig. S3I), consistent with the hypothesis of dedifferentiation wherein cycling progenitors or differentiated cells can regain a MuSC-like phenotype. Because differentiated cells account for a low fraction (15 to 21%) of the CD44^−^ subpopulation (Fig. 3F) and are low cycling (fig. S2A), our results suggest that cycling progenitors are the subpopulation responsible for dedifferentiating into a MuSC-like state. To ensure that changes in CD44 levels were reflective of changes in cluster composition, we measured the gene expression of *PAX7*, *MYOD1*, and *MYOG* in CD44^+^ and CD44^−^ subpopulations at 0 or 7 days after sorting by quantitative reverse transcription polymerase chain reaction (qRT-PCR). Generally, *PAX7* levels decrease over time in the CD44^+^ cells and increased in CD44^−^ cells; *MYOD1* and *MYOG* increased in the CD44^+^ subpopulation and decreased in CD44^−^ cells, indicative of cells changing their myogenic status (fig. S3J).

We wished to determine whether cycling progenitors can directly transition toward differentiated states. We sorted CD44^+^ (MuSC-like) and CD44^−^ cells (which include cycling progenitors, G_1_-phase, S-phase, and differentiated cells; Fig. 3F) and stained them for MyHC 7 days after sorting. Because differentiated and G_1_-phase cells are low cycling, while cycling progenitors and S-phase cells are high cycling (fig. S2A), the low-cycling subpopulations are diluted out of the CD44^−^ panel during the 7 days of incubation. This allows us to directly compare the differentiation potential of CD44^+^ (MuSC-like) and CD44^−^ (cycling progenitors and S-phase) cells. We observed differentiated MyHC^+^ cells predominantly in CD44^+^ and unsorted cells, but not in CD44^−^ cells (Fig. 3G). On the basis of this finding, we hypothesize that, in aRMS, only MuSC-like (CD44^+^) cells can transition toward differentiated states, whereas cycling progenitors (CD44^−^) lack this ability and have to dedifferentiate to MuSC-like cells first before they can enter the differentiation pathway, consistent with the bifurcated trajectory model (Fig. 3A).

In summary, our data show that RMS tumors mirror transcriptional programs of developing or regenerating muscles (Fig. 3H). The aggressive aRMS subtype contains not only at least two self-renewing dynamically interconverting subpopulations, namely, the transcriptionally immature MuSC-like cells and the cycling progenitors, but also a minor subpopulation that exits the cell cycle and undergoes differentiation.

### PAX3::FOXO1 sustains aRMS cells in the MuSC-like/cycling progenitor trajectory loop

**Fig. 4.**
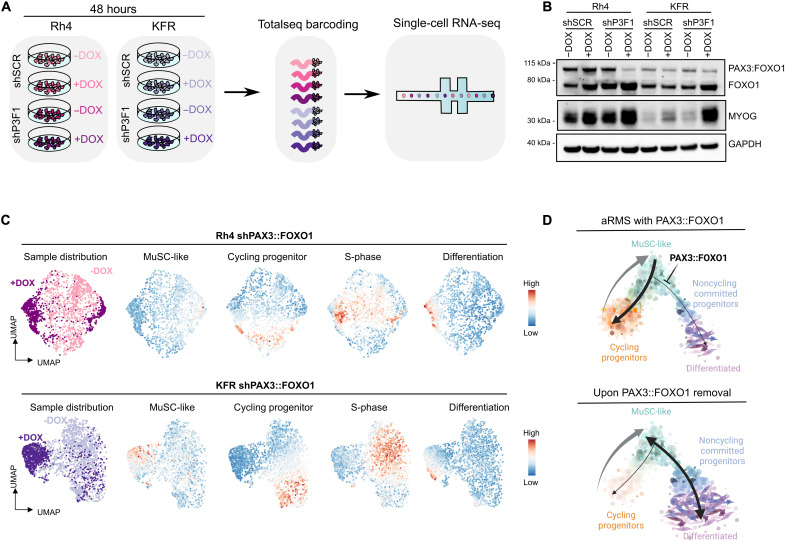
PAX3::FOXO1 down-regulation leads to MuSC-like and differentiated subpopulations. (**A**) Schematic workflow. (**B**) Representative WB of Rh4 and KFR cells cultured with (+DOX) or without (−DOX) DOX for 48 hours. (**C**) UMAP plot of 1978 Rh4 and 2589 KFR cells following KD of PAX3::FOXO1. Lines with shP3F1 were cultured with DOX for 48 hours (+DOX) to induce protein down-regulation and profiled by scRNAseq. Control lines that were not exposed to DOX are also shown (−DOX). UMAP plots are colored by DOX exposure (left) or the overall expression (color scale) of the identified signatures (right). (**D**) Proposed model of PAX3::FOXO1 heterogeneity across aRMS cell lines. Upon PAX3::FOXO1 removal, the differentiation block is released and the oncogenic loop is disrupted. The cycling progenitor subpopulation disappears, and the remaining cells display MuSC-like or differentiated features.

PAX3::FOXO1 is the major driver of aRMS, known to repress myogenic differentiation. To determine how PAX3::FOXO1 regulates the identified subpopulations, we used two aRMS cell lines (Rh4 and KFR) expressing a doxycycline (DOX)–inducible short hairpin RNA (shRNA) directed against PAX3::FOXO1 (shP3F1) or a control short hairpin RNA (shSCR) (*43*). We decreased PAX3::FOXO1 levels by treatment with DOX for 48 hours and measured the transcriptome of Rh4 and KFR cells using droplet-based scRNAseq (Fig. 4A). Upon knockdown (KD) of the fusion protein (Fig. 4B and fig. S4A), both Rh4 and KFR cells underwent consistent transcriptional changes at the global level, including up-regulation of the skeletal muscle genes *MYL1*, *MYOG*, and *DES*, and repression of the known PAX3::FOXO1 downstream targets *STATH*, *PIPOX*, and *ASS1* in Rh4 cells (fig. S4B and table S4). At the single-cell level, KD of the fusion protein induced clear cellular shifts: most of the cells acquired a signature characteristic of differentiated cells, and a minority of the cells acquired a MuSC-like signature; the cycling progenitor and S-phase signatures were lost (Fig. 4C). These shifts were not observed in the control shSCR lines (fig. S4C). Collectively, these results support a model in which PAX3::FOXO1 sustains aRMS cells in the cycling progenitor state. Upon PAX3::FOXO1 KD, cells either undergo differentiation or halt in a MuSC-like state, a finding that can inform the design or implementation of PAX3::FOXO1*-*targeted therapy (Fig. 4D).

### Chemotherapy enriches aRMS tumors for the plastic MuSC-like subpopulation

**Fig. 5.**
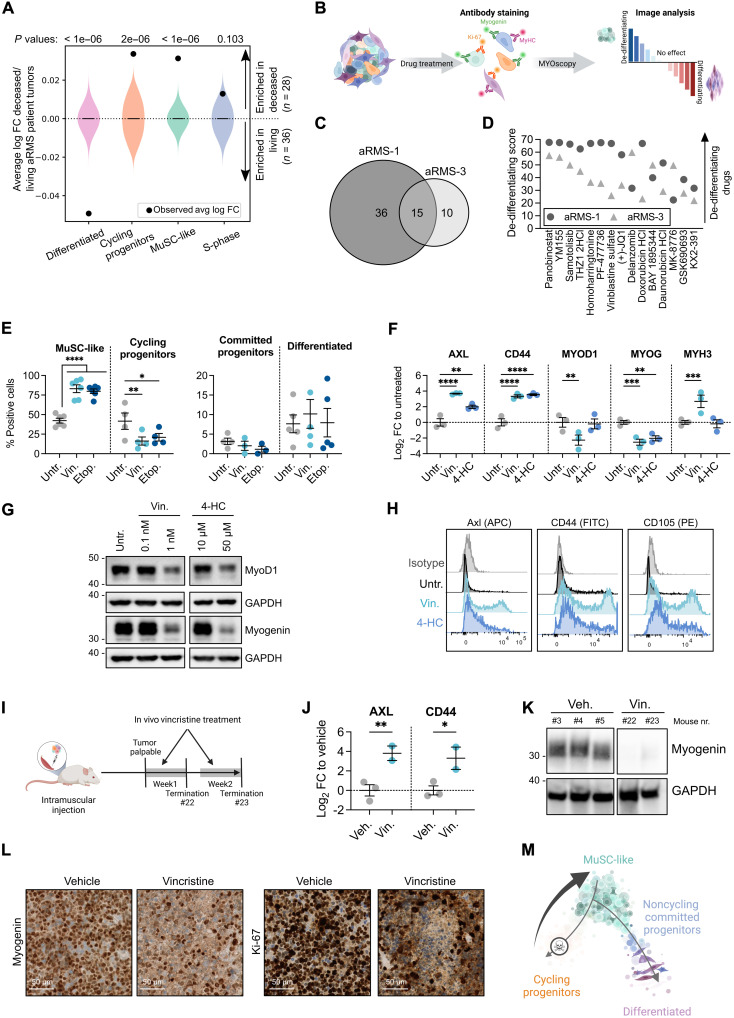
Chemotherapy induces a transition toward MuSC-like states in aRMS. (**A**) Association between cluster-associated signatures and aRMS patient outcome. Violin plots show log fold change distributions between deceased and living patients assuming no association (10^7^ simulation replicates). Black dots represent the calculated associations. (**B**) Experimental workflow. (**C**) Number of dedifferentiating drug hits at 1 μM. (**D**) Shared dedifferentiating drugs at 1 μM ranked on the basis of the average dedifferentiating score. A score of zero represents the baseline score of untreated controls. (**E**) Immunofluorescence quantification of aRMS-1 cells exposed to 10 nM vincristine or 1 μM etoposide for 72 hours; ordinary two-way ANOVA with uncorrected Fisher’s LSD. (**F**) qRT-PCR data generated with aRMS-1 cells exposed to 10 nM vincristine sulfate or 50 μM 4-HC for 48 hours; ordinary two-way ANOVA with uncorrected Fisher’s LSD. (**G**) WB images of aRMS-1 cells exposed to vincristine or 4-HC for 48 hours. GAPDH was used as loading control; samples were loaded on the same gel. (**H**) Representative FACS plots of aRMS-1 cells treated with 1 nM vincristine or 10 μM 4-HC for 48 hours. (**I**) Experimental workflow. (**J**) qRT-PCR data generated with aRMS-1 tumors collected after in vivo treatment with vincristine (10 mg/kg); ordinary two-way ANOVA with uncorrected Fisher’s LSD. (**K**) WB images of aRMS-1 PDX tumors treated in vivo with vincristine. Samples were loaded on the same gel. (**L**) Expression of myogenin and Ki-67 as determined by immunohistochemistry in aRMS-1 PDX tumors following in vivo treatment with vincristine or vehicle. (**M**) Proposed model of treatment selection following chemotherapy in aRMS. *P < 0.05; **P < 0.01; ***P < 0.001; ****P ≤ 0.0001. Data are represented as means ± SEM of the indicated number of biological replicates. Untr., untreated; Vin, vincristine sulfate; 4-HC, 4-hydroperoxycyclophosphamide; Veh, vehicle; FC, fold change.

Our single-cell analysis showed that aRMS tumors contain MuSC-like cells that either become cycling progenitors or commit to differentiation (Fig. 3H). We evaluated the clinical impact of the identified subpopulations by testing whether their transcriptomic signatures are associated with different clinical outcomes in aRMS patients (*35*). Notably, MuSC-like (*P* < 1 × 10^−6^), cycling progenitor (*P* = 2 × 10^−6^), and S-phase (*P* = 0.103) signatures were all highly represented in deceased aRMS patients (*35*), whereas the differentiated (*P* < 1 × 10^−6^) signature was strongly enriched in living aRMS patients (Fig. 5A).

To understand the mechanisms and therapeutic regulators of MuSC-like cell-fate decisions, we directed our efforts toward the identification of (i) drug-induced beneficial effects, which are compounds promoting cellular commitment and differentiation, and (ii) drug-induced detrimental effects, which we defined as compounds that dedifferentiate aRMS into the plastic MuSC-like subpopulation. To quantify the aRMS cellular states in a high-throughput manner, we first established MYOscopy, a high-content microscopy screening platform, which was able to distinguish quiescent (myogenin^−^/Ki-67^−^) and cycling MuSC-like (myogenin^−^/Ki-67^+^), cycling (myogenin^+^/Ki-67^+^/MyHC^−^) and committed progenitors (myogenin^+^/Ki-67^−^/MyHC^−^), and differentiated (MyHC^+^) cellular states (fig. S5, A and B). We exposed two aRMS primary cultures to a drug library of 244 compounds in a dose range of 10 nM to 10 μM and used MYOscopy to quantify the cellular composition after 72 hours of treatment (Fig. 5B and table S5). Fifteen compounds, which included the commonly used chemotherapeutics vinblastine sulfate, daunorubicin, and doxorubicin, consistently enriched both aRMS-1 (IC-pPDX-104) and aRMS-3 (IC-pPDX-35) primary cultures for MuSC-like cells (Fig. 5, C and D).

As resistance to chemotherapy is a major concern in aRMS, we characterized the effect of standard-of-care chemotherapeutics on the cellular composition of aRMS primary cultures by immunofluorescence. After a 72-hour exposure to vincristine or etoposide, we observed a clear decrease in the number of nuclei, a significant increase in the proportion of MuSC-like cells, and a decrease in cycling progenitors; there was no significant difference in the composition of noncycling committed progenitors and differentiated cells (Fig. 5E and fig. S5, C to E). To further validate the results at the transcriptome level, we exposed the same primary cultures to vincristine and 4-hydroperoxycyclophosphamide (4-HC), the active metabolite of cyclophosphamide, and evaluated their effects on the mRNA level of selected myogenic markers. Both drugs increased *AXL* and *CD44* mRNA levels, markers of the MuSC-like subpopulation, and decreased *MYOG*, characteristic for the committed/differentiated subpopulation; vincristine decreased the expression of the activated myogenic marker *MYOD1*, whereas we found no consistent change in the differentiation marker *MYH3* (Fig. 5F and fig. S5F). We confirmed drug-induced down-regulation of MyoD1 and myogenin at the protein level by Western blot (WB) analysis (Fig. 5G and fig. S5, G and H), and up-regulation of the progenitor markers CD44, Axl, and CD105 by flow cytometry analysis (Fig. 5H and fig. S5I).

To determine the effect of vincristine treatment in vivo, we injected aRMS-1 (IC-pPDX-104) cells orthotopically into immunodeficient NSG mice and collected the tumors at 1 and 2 weeks after vincristine treatment (Fig. 5I). In vivo treated tumors showed increased mRNA expression of the MuSC-like markers *CD44* and *AXL* (Fig. 5J) and decreased protein expression of myogenin (Fig. 5K). Immunohistochemical staining of the tumors confirmed a decrease in myogenin^+^ and Ki-67^+^ cells following treatment (Fig. 5L).

We hypothesized that MuSC-like aRMS cells are either intrinsically resistant to treatment, as described for eRMS (*36*), or that chemotherapy rewires aRMS trajectories toward MuSC-like states. We assessed the in vitro efficacy of vincristine and 4-HC on sorted CD44^+^ and CD44^−^ cells (fig. S5J) and found that both drugs were equally effective on the two subpopulations, with no significant difference in their median inhibitory concentration (IC_50_) values (fig. S5, K and L). These results suggest that chemotherapy enriches aRMS cells for the MuSC-like state (Fig. 5M), most likely by rewiring the cellular trajectory and not necessarily by selecting intrinsically chemoresistant subpopulations.

### Trametinib hijacks the cell fate of MuSC-like aRMS toward differentiation

**Fig. 6.**
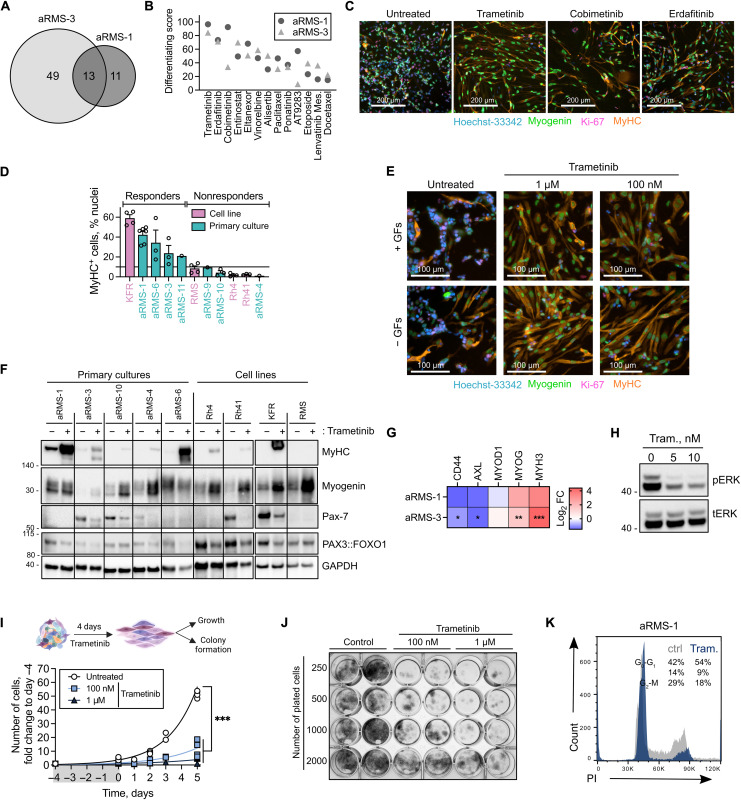
Image-based high-content drug screening identifies the MEK inhibitor trametinib as a potent inducer of myogenic differentiation in aRMS primary cultures. (**A**) Number of drug hits promoting differentiation in aRMS-1 and aRMS-3 at 1 μM. (**B**) Shared differentiating drugs at 1 μM ranked on the basis of the average differentiating score across aRMS-1 and aRMS-3 cells. (**C**) Representative images of aRMS-1 cells following 72 hours of drug treatment at 1 μM. (**D**) Percentage of differentiated (MyHC^+^) cells following treatment with 100 nM trametinib for 72 hours. Data are represented as means ± SEM of the indicated number of biological replicates. A threshold of 10% was used to separate responders from nonresponders. (**E**) Representative images of aRMS-1 cells following 72 hours of drug treatment with trametinib in the presence (+GFs) or absence (−GFs) of the growth factors bFGF and EGF. (**F**) WBs of aRMS primary cultures and cell lines exposed to 50 nM trametinib for 96 hours. (**G**) qRT-PCR data of aRMS-1 and aRMS-3 cells exposed to 50 nM trametinib for 96 hours. Data are represented as means ± SEM of *n* ≥ 2 biological replicates; multiple unpaired *t* tests. (**H**) WB analysis of phosphorylated ERK in aRMS-1 cells after exposure to trametinib for 3 hours. (**I**) Proliferation curve of aRMS-1 cells exposed to trametinib for 4 days (gray bar) and cultivated in drug-free medium for further 5 days, as determined by cell counting; ordinary two-way ANOVA with Dunnett’s correction. (**J**) Colony-forming ability of aRMS-1 cells exposed to trametinib for 4 days and seeded at the indicated cell densities in duplicates in drug-free medium for 10 days. (**K**) Representative cell cycle analysis plot of aRMS-1 cells exposed to 25 nM trametinib for 96 hours and stained with propidium iodide. *P < 0.05; **P < 0.01; ***P < 0.001.

Differentiation therapy has been proposed as a promising therapeutic strategy in cancers with dysregulated development (*23*, *24*). We therefore reanalyzed our drug screening (Fig. 5B) and focused on identifying compounds that hijacked aRMS MuSC-like cells toward the noncycling committed progenitor–differentiated trajectory while simultaneously decreasing cycling progenitors. Of the 244 anticancer agents tested, 72 compounds promoted differentiation in aRMS primary cultures; 13 of the 72 compounds (18%) were effective on both aRMS-1 (IC-pPDX-104) and aRMS-3 (IC-pPDX-35) cells (Fig. 6A). These compounds included the mitogen‑activated protein kinase kinase (MEK)inhibitors trametinib and cobimetinib, the fibroblast growth factor receptor (FGFR) inhibitor erdafitinib, the vascular endothelial growth factor receptor (VEGFR)/FGFR inhibitors ponatinib and lenvatinib mesylate, and the aurora kinase inhibitors alisertib and AT9283, among others (Fig. 6B). Trametinib, cobimetinib, and erdafitinib appeared as top-differentiating compounds, and all induced morphologically robust myogenic differentiation (Fig. 6C).

Notably, in contrast to our finding using aRMS primary cultures, a recent report failed to detect differentiation in aRMS cell lines treated with trametinib (*26*). We therefore compared the therapeutic potential of trametinib-induced differentiation in *n* = 7 primary cultures versus *n* = 4 cell lines. Trametinib shifted the primary cultures toward noncycling committed progenitor and differentiated states while reducing the cycling progenitors, starting from exposures to 10 nM (fig. S6, A and B). In cell lines, the effect was generally smaller (fig. S6C). We uncovered patient-specific responses to trametinib (Fig. 6D), which were independent from culturing conditions and from the presence of growth factors (Fig. 6E). Both primary cultures and cell lines exposed to trametinib showed increased myogenin protein levels and decreased Pax-7 and PAX3::FOXO1, while the increase in MyHC expression was predominantly observed in primary cultures (Fig. 6F and fig. S6D). In primary cultures, trametinib decreased mRNA expression of *CD44* and *AXL*, while it increased *MYOG* and *MYH3*, consistent with cellular shifts toward differentiated states (Fig. 6G). We confirmed trametinib on-target effects by measuring extracellular signal–regulated kinase (ERK) phosphorylation following treatment. In all tested primary cultures and cell lines, we observed a decrease in phosphorylated ERK upon exposure to low nanomolar doses of trametinib, which correlated with the observed trametinib-induced differentiation (*R*^2^ = 0.83; *P* = 0.03; *n* = 5; Fig. 6H and fig. S6, E and F).

To investigate whether differentiated aRMS cells retain their malignant potential, we measured the proliferation and colony-forming ability of aRMS-1 (IC-pPDX-104) cells after a 4-day exposure to various concentrations of trametinib (Fig. 6I). Trametinib showed a dose-dependent reduction of cell proliferation and impairment of colony formation, with small effects at doses as low as 10 nM and larger effects at higher doses (Fig. 6, I and J, and fig. S6, B and G). Cell cycle analysis further revealed that trametinib treatment led to an arrest in G_0_-G_1_ phase, as expected from cells undergoing terminal myogenic differentiation (Fig. 6K and fig. S6H). Together, these findings underscore the advantage of using primary cells rather than cell lines, and suggest MEK inhibition as a cell-fate determinant and therapeutic vulnerability of aRMS tumors.

### RAF inhibitors enhance trametinib-induced aRMS differentiation and inhibit in vivo PDX growth

**Fig. 7.**
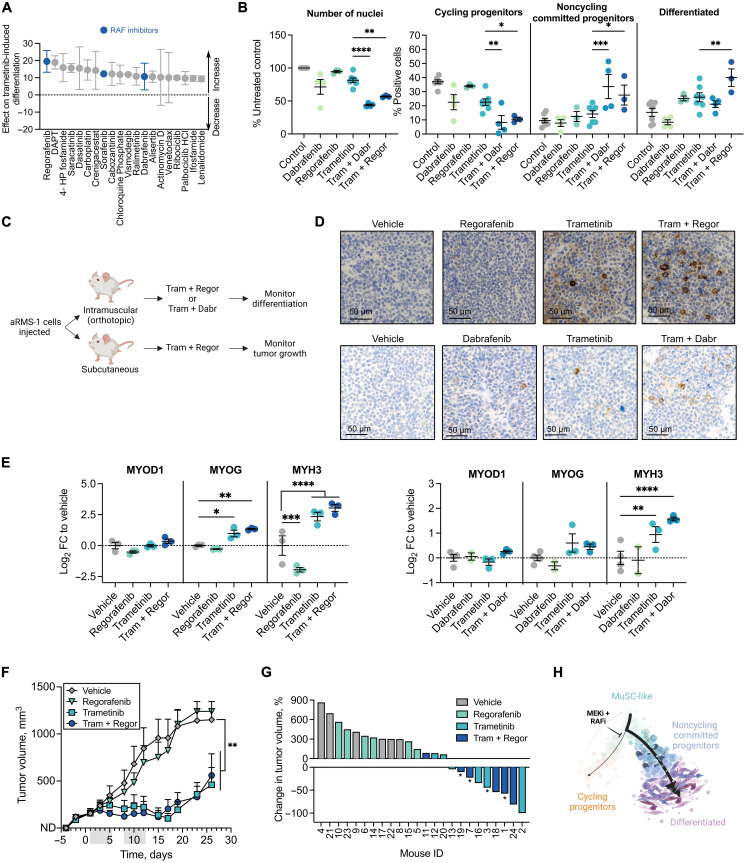
Vertical inhibition of the RAF-MEK-ERK cascade potentiates trametinib-induced differentiation and inhibits aRMS tumor growth. (**A**) Top 20 trametinib-potentiating drugs ranked on the basis of their effect on trametinib-induced differentiation. A score of zero represents the baseline score of trametinib alone; drugs with a positive score potentiate trametinib-induced differentiation. *n* = 2 biological replicates. (**B**) Quantification of immunofluorescence analysis of aRMS-1 cells exposed to vehicle controls, 10 μM dabrafenib (Dabr), 1 μM regorafenib (Regor), 10 nM trametinib (Tram), or the indicated combinations for 72 hours; ordinary two-way ANOVA with uncorrected Fisher’s LSD. (**C**) Schematic of in vivo validation experiment. (**D**) Expression of MyHC as determined by immunohistochemistry in aRMS-1 PDX tumors following in vivo treatment with trametinib (5 mg/kg), regorafenib (15 mg/kg), or their combination (top row) or with trametinib (1 mg/kg), dabrafenib (15 mg/kg), or their combination (bottom row). (**E**) qRT-PCR analysis of aRMS-1 PDX tumors; ordinary two-way ANOVA with uncorrected Fisher’s LSD. (**F**) Monitoring of tumor growth in mice that were injected with aRMS-1 cells and treated with vehicle, trametinib (5 mg/kg), regorafenib (15 mg/kg), or their combination for two cycles (gray bars); ordinary two-way ANOVA with Dunnett’s multiple comparison correction. (**G**) Waterfall plot showing the change in tumor volume in mice treated with vehicle, trametinib (5 mg/kg), regorafenib (15 mg/kg), or their combination, at the end of the treatment period (day 12). Mice marked with “*” had to be euthanized before the treatment end point due to toxicity. (**H**) Proposed model of trajectory rewiring in aRMS following treatment with the MEK inhibitor (MEKi) trametinib in combination with the RAF inhibitor (RAFi) regorafenib or dabrafenib. *P < 0.05; **P < 0.01; ***P < 0.001; ****P ≤ 0.0001. Data points are represented as means ± SEM of the indicated number of biological replicates.

Because clinical trials with single-targeted agents have been largely unsuccessful, we next sought to identify trametinib-potentiating drugs by performing a combinatorial screening in aRMS-1 (IC-pPDX-104) cells. We used a library of 243 compounds either alone or in combination with 50 nM trametinib and measured differentiation using MYOscopy (table S6). We found 97 drugs that potentiated trametinib-induced differentiation; of the top 20 hits, we identified several RAF inhibitors (Fig. 7A). These included regorafenib, a kinase inhibitor currently in clinical trial in RMS patients (NCT01900743), which acts as multikinase inhibitor and, besides mutant and wild-type B-RAF and RAF-1, also targets KIT, PDGFRB, RET, and VEGFR1-3 (*44*), its derivatives sorafenib, a multikinase inhibitor of RAF-1 and B-RAF (*45*), and dabrafenib, a selective inhibitor of mutant B-RAF and to a minor extent of wild-type B-RAF (*46*). Dabrafenib is currently already used in combination with trametinib in patients with melanoma (*47*) and non–small cell lung cancer (*48*). We measured the differentiating effect of trametinib-dabrafenib and trametinib-regorafenib combinations at different drug ratios and observed a synergistic score when 10 nM trametinib was combined with 1 μM regorafenib or 10 μM dabrafenib (fig. S7A). At this drug ratio, both combinations significantly decreased the number of nuclei and of cycling progenitors and shifted aRMS cells toward more noncycling committed progenitor and differentiated states compared to single trametinib treatment (Fig. 7B and fig. S7B). To validate these findings in in vivo PDX models, we injected aRMS-1 (IC-pPDX-104) cells orthotopically and subcutaneously and measured the effect of a 2-week drug treatment on tumor differentiation and growth inhibition (Fig. 7C and table S7). We applied drug concentrations that were in the range of the maximum human tolerated doses (table S8). Tumors that were exposed to trametinib or to its combinations with regorafenib or dabrafenib displayed higher abundance of myogenin^+^ and MyHC^+^ cells and less of Ki-67^+^ cells (Fig. 7D and fig. S7C). Tumors that were exposed to the combinations also showed higher mRNA levels of the differentiation markers *MYOG* and *MYH3* but not of the myoblast marker *MYOD1* (Fig. 7E), indicative of cells undergoing myogenic differentiation. Last, we examined the effect of trametinib-regorafenib combinatorial treatment on aRMS tumor growth and observed significantly impaired tumor growth in mice that were treated with trametinib or with the combination (Fig. 7, F and G). Overall, these data suggest that trametinib-induced suppression of the RAS pathway in combination with RAF inhibition is a feasible treatment strategy in aRMS that targets cellular differentiation (Fig. 7H).

## DISCUSSION

Single-cell RNA and protein profiling are powerful methods for investigating developmental heterogeneity and plasticity in human cancers. Here, we leveraged scRNAseq and CyTOF to analyze clinically relevant models of RMS and to dissect the cellular, molecular, and functional intratumoral heterogeneity. Our findings are in agreement with two recent publications (*36*, *49*), providing robust evidence in support of the hypothesis that both aRMS and eRMS tumors harbor developmentally halted cells with myogenic potential that fail to differentiate. Although the different studies provide no consensus on the nomenclature used to define RMS subpopulations [“mesoderm,” “myoblast,” and “myocyte” in (*36*); “mesenchymal-like,” “proliferation,” and “muscle” in (*49*); “MuSC-like,” “cycling progenitors,” and “differentiated” in our work], similar gene signatures suggest that the compartments are conceptually the same. A meta-analysis integrating the different RMS scRNAseq datasets will be needed to confirm this hypothesis.

In our study, we identify a myogenin^+^ cycling progenitor cell state that is only transiently found in myogenesis during muscle development and regeneration (*32*) and that characterizes the more aggressive fusion-positive aRMS subtype. Myogenin is typically a hallmark transcription factor of a nonproliferative myogenic commitment state that drives expression of differentiation markers, such as MyHCs. In aRMS, the cycling progenitor state continues to proliferate, although it has initiated myogenic differentiation; hence, it could serve as a cellular target for aRMS differentiation therapy. In addition, we identify an RMS subpopulation resembling early MuSCs (MuSC-like) and one resembling differentiated muscle cells. While these cellular states were clearly present in primary cultures, O-PDXs, and patient samples, their abundance was notably decreased in cell lines, providing support for the preclinical use of primary cultures in lieu of cell lines.

An emerging body of evidence suggests that childhood tumors contain developmental hierarchies (*6*, *7*, *50*). Here, we provide evidence that this phenomenon is a characteristic of RMS. Moreover, a characterization of the developmental hierarchies in the aggressive aRMS models reveals a distinction of relevance to clinical outcome. When MuSC-like cells become aberrant cycling progenitors, they correlate with worst patient prognosis; on the other hand, if they commit to a noncycling committed/differentiated cell state, they drive better patient prognosis. Our data support an aRMS tumor model in which cycling progenitors and MuSC-like cells coexist as dynamically interconverting plastic states, fueling a continuous oncogenic loop that must be therapeutically disrupted. A similar transition from mesenchymal-like to differentiated states has been identified by lineage-tracing experiments on eRMS (*49*). However, the authors did not detect a reversed switch from the differentiated state to mesenchymal-like state in eRMS (*49*), which could be a unique characteristic of the aggressive aRMS subtype. Our results are in line with a previous report that failed to detect a cancer stem cell (CSC) subpopulation with self-renewing and tumor-initiating properties in aRMS (*51*), rather suggesting a role for cellular plasticity instead of the classical CSC hypothesis at the root of this tumor resistance and aggressiveness.

Our results demonstrate that following genetic perturbation of the fusion protein PAX3::FOXO1, cells undergo myogenic differentiation, as previously described (*35*, *52*), but they also revert back to a MuSC-like state. These findings suggest that PAX3::FOXO1 maintains aRMS tumor cells in the MuSC-like*/*cycling progenitor trajectory loop. The other cellular states may have intrinsically lower PAX3::FOXO1 expression or may be less dependent on expression of the fusion protein itself. However, it still remains to be determined whether a subpopulation of cells that is completely independent of PAX3::FOXO1 exists and whether it has the potential to proliferate, as suggested based on data from ectopic models (*53*). Both mesenchymal stem cells (*13*) and differentiated myogenic cells (*15*) have previously been described as possible cells of origin for aRMS.

Patients with aRMS are known to respond to first-line treatments, but they often relapse (*19*), suggesting that the therapy fails to eliminate all tumor cells. Unlike eRMS in which the mesoderm/MuSC-like subpopulation has been regarded as resistant to treatment (*36*), our findings suggest that MuSC-like aRMS cells are not intrinsically chemoresistant. On the basis of our data, chemotherapy induces a temporary and reversible phenotype switch, by dedifferentiating aRMS cells into a drug-tolerant MuSC-like state, a phenomenon that has been described for other cancers (*54*, *55*). It is this state that allows aRMS cells to survive the treatment, to reexpand, and to recapitulate the trajectories that reflect the tumor complexity, leading to relapse. These observations pinpoint reprogramming and cellular plasticity as emerging drug resistance mechanisms in aRMS, as suggested for other cancer types (*56*, *57*).

The presence of differentiated RMS cells under basal conditions indicates that some cells overcome the differentiation block and that RMS tumors may be amenable to differentiation therapy. The potential benefits of such a strategy are highlighted by the strong statistical association between the differentiation signature and aRMS patient survival. To determine whether we could pharmacologically capitalize on this process, we screened for compounds that promote differentiation. The image-based screening approach we developed here for aRMS can serve as a guide for other cancers to better understand the determinants of cancer cell–fate decisions. We identified trametinib-induced MEK inhibition as a strategy to rewire the tumor hierarchy in aRMS primary cell cultures, in a manner not seen with aRMS cell lines by us or others (*26*). This leads us to conclude that cell lines may be less prone to surmount the differentiation block and therefore lack utility for drug screening. Our findings underscore the use of primary cells as models to study RMS tumorigenesis.

Our combination screen identified the multikinase inhibitors regorafenib, sorafenib, and dabrafenib among the top trametinib-potentiating compounds in aRMS. Although regorafenib and sorafenib target angiogenic (VEGFR), stromal [platelet-derived growth factor receptor (PDGFR)], and oncogenic kinases (KIT and RAF), dabrafenib selectively inhibits RAF kinases (*44*–*46*). We therefore conclude that RAF + MEK inhibition leads to myogenic differentiation in aRMS, in line with a recent study on eRMS (*25*), showing that vertical inhibition of the RAF-MEK-ERK cascade effectively induces differentiation. We were able to confirm our in vitro findings by inducing myogenic differentiation and potently suppressing tumor growth in an aRMS PDX model with an in vivo combination of RAF (regorafenib or dabrafenib) and MEK (trametinib) targeting at clinically relevant doses. Clinical studies with the RAF inhibitor regorafenib are currently ongoing in RMS (NCT01900743), while studies with the MEK inhibitor cobimetinib are underway in eRMS patients (NCT02639546). On the basis of our findings, we expect that RAF inhibitors, such as regorafenib, would benefit from a combination with MEK inhibitors, an approach that could lead to increased aRMS cellular differentiation. Notably, the use in the clinic of differentiation therapy for aRMS and its combination with chemotherapy have to be carefully evaluated, as our work suggests that they induce opposite lineage shifts. To be successful, one would first need to eliminate the rapidly dividing cycling progenitors with chemotherapy and only then “mobilize” the remaining MuSC-like cells to cause their exhaustion using differentiating agents.

In summary, our work provides a comprehensive single-cell transcriptomic and proteomic atlas of RMS. This atlas resolves the cellular and functional heterogeneity of RMS and reveals key cellular and molecular signatures, with therapeutic implications for chemoresistance and tumor relapse. Our study sheds light on the cellular fate mechanisms underlying impaired differentiation in the aggressive aRMS subtype and provides opportunities to therapeutically restore myogenic differentiation and block tumor growth.

## MATERIALS AND METHODS

### Patient-derived xenografts

All animal experiments were conducted under license of the authorities in compliance with the national laws and regulations and approved by the Charité University Medicine, the ethics committee of the Institut Curie CEEA-IC #118 (authorization APAFIS#11206-2017090816044613-v2 given by National Authority), the Institut Curie institutional review board (OBS170323 CPP ref. 3272; n_ de dossier 2015-A00464-45), and the Zürich canton government (license number ZH013/2021). The PDXs used in this study were generated from patient biopsies collected at St. Jude Children’s Research Hospital, University Children’s Hospital Zurich, Institut Curie Paris and Charité, and University Hospital Berlin. All patients gave written informed consent at the participating institutions. Patient characteristics and information on the clinical status can be found in table S1.

PDXs were generated as previously described (*58*, *59*): In short, patient biopsies were first expanded in immunodeficient NSG, Janvier Rj:NMRI-*Foxn1^nu/nu^*, or Taconic NOD.Cg-*Prkdc^scid^ Il2rg^tm1Sug^*/JicTac mice. Tumors were isolated from mice when reaching a size of 700 to 1300 mm^3^, mechanically minced into smaller pieces using scalpels, and retransplanted in secondary recipient mice. To generate single-cell suspensions, PDX tumors were mechanically and enzymatically digested using liberase DH (200 μg/ml; Roche, 5401054001) and 1 mM MgCl_2_ in 1× Hanks’ balanced salt solution buffer (Sigma-Aldrich, H6648) for 30 to 60 min at 37°C. Cell suspension was filtered through a 70-μm cell strainer to remove remaining tumor pieces, washed with phosphate-buffered saline (PBS), and used immediately to produce cultured cells or frozen in freezing medium CryoStor CS10 (STEMCELL Technologies, #07930).

For in vivo drug testing, 1 million to 3 million aRMS-1 (IC-pPDX-104) cells were first expanded in vitro for a low number of passages and injected orthotopically into the gastrocnemius muscle (to assess the tumor composition) or subcutaneously into the flank (to assess the tumor volume) of 6- to 10-week-old NSG mice. When tumors became palpable (average tumor size reached 100 to 200 mm^3^), mice were randomized in treatment and vehicle-treated control cohorts. For the trametinib-dabrafenib combination experiment, drugs were dosed as follows: trametinib (1 mg/kg; Selleckchem, S2673) dissolved in PBS with 1% dimethyl sulfoxide (DMSO; Sigma-Aldrich, D8418) and administered by intraperitoneal injection five times a week and dabrafenib (15 mg/kg; Selleckchem, S2807) dissolved in PBS with 5% DMSO (Sigma-Aldrich, D8418) and administered by oral gavage five times a week. For the trametinib-regorafenib combination, drugs were dosed as follows: trametinib (5 mg/kg; Selleckchem, S2673) dissolved in PBS with 5% DMSO (Sigma-Aldrich, D8418) and administered by intraperitoneal injection five times a week and regorafenib (15 mg/kg; Selleckchem, S2807) dissolved in double-distilled water with 5% DMSO, 30% polyethylene glycol 300 (Sigma-Aldrich, 90878), and 5% Tween 80 (Sigma-Aldrich, P4780) and administered by oral gavage five times a week. Mice were euthanized at the end of the second treatment week. Tumors were harvested 4 hours after the last treatment and processed for qRT-PCR and immunohistochemistry. For vincristine treatment, mice were treated with vincristine sulfate (10 mg/kg; MedChemExpress, HY-N0488) dissolved in PBS and administered by intraperitoneal injection two times a week (Monday and Thursday). A mouse treated with vincristine was euthanized during treatment due to severe body weight loss (>20% than baseline) and was therefore excluded from the analysis; the other *n* = 2 mice were euthanized at the end of the first and second week after the start of the treatment. Tumor volume was measured three times a week using a caliper. Mice were euthanized when tumor volumes reached 1000 mm^3^.

### Primary cultures

To produce primary cultures, dissociated PDX tumors were grown on plates coated with Matrigel (Corning, 354234) diluted 1:10 in Advanced DMEM (Dulbecco’s modified Eagle’s medium)/F-12 medium (Thermo Fisher Scientific, 12634010) and left at room temperature for 30 to 60 min to solidify. Cells were cultured in Advanced DMEM/F-12 (Thermo Fisher Scientific, 12634010) medium supplemented with penicillin/streptomycin (100 U/ml; Thermo Fisher Scientific, 15140122), 2 mM GlutaMAX (Life Technologies, 335050-061), 0.75× B-27 (Thermo Fisher Scientific, 17504044), basic fibroblast growth factor (bFGF) (20 ng/ml; PeproTech, AF-100-18B), and epidermal growth factor (EGF) (20 ng/ml; PeproTech, AF-100-15) (“Complete F12” medium) or in Neurobasal medium (Thermo Fisher Scientific) supplemented with penicillin/streptomycin (100 U/ml; Thermo Fisher Scientific, 15140122), 2 mM GlutaMAX (Life Technologies, 335050-061), and 2× B-27 (Thermo Fisher Scientific, 17504044) (“Complete NB” medium). In some cases, complete NB medium was supplemented with bFGF (20 ng/ml; PeproTech, AF-100-18B) and EGF (20 ng/ml; PeproTech, AF-100-15). For further passaging, cells were washed with PBS and detached with Accutase (Sigma-Aldrich, A6964) diluted 1:2 to 1:3 in PBS. Information for each model can be found in table S9. All RMS primary cultures were regularly tested to ensure no mycoplasma contamination with the LookOut Mycoplasma PCR-Detection Kit (Sigma-Aldrich, MP0035-1KT).

### Cell lines

The cell lines Rh4 (RRID: CVCL_5916) and Rh41 (RRID: CVCL_2176, both provided by P. Houghton, Greehey Children’s Cancer Research Institute, San Antonio, TX), KFR (RRID: CVCL_S637, provided from J. Cinatl, Abteilung für paediatrische Tumor und Virusforschung, Frankfurter Stiftung für krebskranke Kinder, Frankfurt), and RMS (*60*) (provided from J. Shipley, Sarcoma Molecular Pathology, The Institute of Cancer Research) were cultured on uncoated plates in DMEM (Sigma-Aldrich, D5671) supplemented with 10% fetal bovine serum (Life Technologies), penicillin/streptomycin (100 U/ml; Thermo Fisher Scientific, 15140122), and 2 mM GlutaMAX (Life Technologies, 335050-061). For further passaging, cells were washed with PBS and detached with trypsin (BioConcept, 5-51F00-I). All cell lines were authenticated by short tandem repeat (STR) analysis profiling and regularly tested to ensure no mycoplasma contamination with the LookOut Mycoplasma PCR-Detection Kit (Sigma-Aldrich, MP0035-1KT).

### DOX-inducible PAX3::FOXO1 KD

Rh4 and KFR cells containing DOX-inducible shRNAs directed against PAX3::FOXO1 (shP3F1) or against a control hairpin (shSCR) were previously established as described (*43*). shRNA expression was induced using DOX (50 ng/ml) in Rh4 cells and DOX (10 ng/ml) in KFR cells.

### Sample preparation for scRNAseq

Dissociated PDX tumor cells were cultured as primary cultures for a low number of passages before sequencing (table S9). To study single-cell responses upon PAX3-FOXO1 down-regulation, we induced shSCR or shP3F1 expression in Rh4 and KFR cells for 48 hours as described above. For sequencing, cells were detached from the plates and washed once with PBS. Cells from different primary cultures or cell lines or experimental conditions were independently stained with a different oligonucleotide-tagged antibody (TotalSeq-B hashtag antibodies) according to the manufacturer’s protocol and pooled together in equal proportion before sequencing in a single lane (table S9). Briefly, 1 million to 2 million cells were resuspended in 50 μl of Cell Staining Buffer (BioLegend, 420201) and blocked with 5 μl of Human TruStain FcX (BioLegend, 422301) for 10 min at 4°C. Cells were then stained with 1.5 μg of TotalSeq-B hashtag antibodies (BioLegend, 394631, 394633, 394635, 394637, 394639, 394641, 394643, and 394645) in a total volume of 100 μl of Cell Staining Buffer (BioLegend, 420201). After 30-min incubation on ice, cells were washed three times, pooled together, and resuspended in medium at a final concentration of 1000 cells/μl. Cells were stained with trypan blue and counted manually with a hemocytometer to determine their concentration. Viability was confirmed to be >85% before loading onto chip.

### Library preparation and sequencing

Cells were processed for library preparation according to the 10X Genomics Chromium Single Cell 3′ v3 workflow. Cell volume was adjusted to yield a recovery of ~10,000 cells and loaded onto the 10X Genomics Single Cell A Chip. Library quality and concentration were assessed using High-Sensitivity D5000 ScreenTape (Agilent) and then sequenced on the Illumina NovaSeq 6000 System according to 10X Genomics recommendations.

### scRNAseq hashtag sample demultiplexing

Illumina sequencing reads were processed (demultiplexing features and cellular barcodes, read alignment to the reference human genome GRCh38, and gene expression matrix generation) using the 10X Genomics Cell Ranger software Suite (version 3.0.1). Each gene expression matrix was then analyzed independently using Seurat R package (version 3.2.2, *29*) in R (version 3.6.1), with some modifications to the standard pipeline. We first demultiplexed gene expression matrices based on hashtag oligonucleotide (HTO) enrichment (*61*). To do so, we added HTO data as a new independent assay to the RNA data and used centered log-ratio transformation to normalize HTO raw counts. Cells were demultiplexed using Seurat’s HTODemux function with default parameters. We assigned sample identities on the basis of the maximal HTO signal and on the antibody sequence used for staining (table S9).

### scRNAseq data analysis

Demultiplexed samples were analyzed independently. Low-quality cells, identified as cells with <200 or >8000 genes, total number of transcripts [unique molecular identifiers (UMIs) <1000 or >50,000, and/or percentage UMIs mapping to mitochondrial genes >15%, were removed. After log-normalizing the data, we assigned cell cycle scores per single cell, using Seurat’s CellCycleScoring function, and scaled the expression of each gene regressing out the number of UMIs and the percentage UMIs mapping to mitochondrial genes. We then performed principal components analysis (PCA) to denoise the data and, based on elbow plots and the percentage of explained variance, selected the number of principal components (PCs) to consider for downstream analysis. We then built a shared nearest neighbor graph and used the Louvain algorithm for clustering (resolution of 0.2 to 0.3) the cell subpopulation. Single cells were visualized as uniform manifold approximation and projection (UMAP) plots. Differential expression analysis was performed using Seurat’s FindAllMarkers function only considering genes with >log_2_(0.25) fold change and expressed in at least 5% of cells in the cluster. We annotated each cluster in the different datasets independently. Last, to distinguish high-cycling from low-cycling cells, we used S and G_2_-M phase scores calculated by the CellCycleScoring function. High-cycling cells were defined as cells with high S or G_2_-M scores (>0), and low-cycling cells as the ones with low S and G_2_-M scores (<0).

### scRNAseq dataset integration

The scRNAseq datasets derived from *n* = 17 primary RMS cultures and *n* = 3 RMS cell lines were first reduced with reciprocal PCA (RPCA) and integrated with the FindIntegrationAnchors function in Seurat. scRNAseq datasets from individual RMS subtypes or models (*n* = 5 aRMS primary cultures, *n* = 9 eRMS primary culture, and *n* = 3 aRMS cell lines) were integrated individually using SCTransform function followed by the FindIntegrationAnchors function in Seurat.

To compare heterogeneity across different preclinical RMS models, scRNAseq data generated in our study from primary cultures and cell lines were integrated with scRNAseq and single-nuclei RNA-seq (snRNAseq) data generated by Patel *et al.* (*36*) (table S10) from O-PDXs and patient tumors [Gene Expression Omnibus (GEO): GSE174376]. For this purpose, only O-PDX cells of human origin and patient tumor cells that were classified as malignant based on copy number alterations in the original publication were integrated with our scRNAseq data using RPCA and the FindIntegrationAnchors function in Seurat.

To infer the developmental origins of RMS, we reanalyzed a publicly available scRNAseq atlas of human skeletal muscles during embryonic, fetal, and adult muscle development (GEO: GSE147457) (*42*). We integrated cells of muscle origin, as inferred in the original publication, with our *n* = 5 aRMS and *n* = 9 eRMS primary culture scRNAseq data using RPCA and the FindIntegrationAnchors function in Seurat.

To integrate aRMS primary cultures with regenerating muscle cells, we only selected MuSC-like, cycling progenitors, S-phase, and differentiated clusters from our integrated *n* = 5 aRMS primary culture scRNAseq dataset. We then reanalyzed a publicly available mouse single-cell dataset of regenerating muscle (GEO: GSE143437) (*32*), converted mouse genes into human orthologs using the biomaRt R package (*62*), and integrated the data with our aRMS primary cultures using RPCA and the FindIntegrationAnchors function in Seurat. For visualization, the integrated dataset was visualized with PHATE (*t* = 30, k-nearest neighbors (*knn)* = 20) (*41*).

### scRNAseq pseudotime trajectory analysis

Pseudotime ordering of aRMS or eRMS was performed on a subset of cells labeled as MuSC-like, cycling progenitors, S-phase, and differentiated from the integrated aRMS or eRMS primary culture datasets. We reduced and visualized the combined datasets using PHATE (*41*) (*t* = 50, *knn* = 20) instead of UMAP, and used the slingshot package (*40*) to organize cells in pseudotime and infer a trajectory. We set MuSC-like cells as the starting cluster for the trajectory calculation.

### shPAX3::FOXO1 scRNAseq analysis

After hashtag demultiplexing as described above, samples derived from Rh4 or KFR cells were merged and analyzed independently. We first log-normalized the data and then assigned myogenic program scores using the gene signatures previously identified for MuSC-like, cycling progenitors, S-phase, and differentiated clusters using Seurat’s FindAllMarkers function on the integrated RMS dataset (table S2). We then scaled the expression of each gene regressing out the number of UMI and the percentage of mitochondrial genes. We then performed PCA to denoise the data and, based on elbow plots, selected the number of PCs to retain for downstream analysis. We then visualized single cells as UMAP plots. To identify genes differentially expressed upon PAX3::FOXO1 down-regulation, we used Seurat’s FindMarkers function across shPAX3::FOXO1 cells cultured in the presence or absence of DOX (table S4).

### Antibody conjugation with metal isotopes for mass cytometry

Purified antibodies were conjugated to the indicated metals for mass cytometry analysis using a MaxPAR X8 antibody labeling kit (Fluidigm) according to the manufacturer’s instruction. Following labeling, antibodies were diluted in Candor PBS Antibody Stabilization solution (Candor Bioscience GmbH, Wangen, Germany) to 0.2 mg/ml and stored long term at 4°C. Each antibody clone and lot was titrated to optimal staining concentrations using human myoblasts.

### Mass cytometry sample preparation and staining

Cells from primary cultures (table S9) were first detached from plates and pulsed with IdU (Sigma-Aldrich, I7125) at a final concentration of 50 μM for 30 min at 37°C. Dead cells were stained using cisplatin as previously described (*63*). After washing with PBS, cells were resuspended in serum-free DMEM, and cisplatin (Sigma-Aldrich, P4394) was added for 1 min at a final concentration of 25 μM at room temperature. Reaction was quenched by adding 3 ml of DMEM with 10% fetal bovine serum. Cells were then centrifuged at 1200 rpm for 10 min at 4°C, resuspended in cell staining medium [CSM; PBS with 0.5% bovine serum albumin (BSA, Sigma-Aldrich, A8022) and 0.02% sodium azide], and fixed with 1.6% paraformaldehyde (PFA; Thermo Fisher Scientific, 28908) for 10 min on ice. Cells were washed with CSM and stained with antibodies against surface markers included in the mass cytometry panel for 1 hour at room temperature. Cells were washed twice with CSM and permeabilized with methanol for 10 min on ice. Cells were washed twice with CSM and stained with antibodies against intracellular markers included in the mass cytometry panel for 1 hour at room temperature. Cells were washed twice with CSM and stained with 1 ml of 191/193Ir DNA intercalator (Fluidigm) diluted in PBS (1:5000) with 1.6% PFA for 20 min at room temperature.

### Mass cytometry measurement

Cells were acquired on the CyTOF2 mass cytometer (Fluidigm) at an event rate of approximately 500 cells per second as previously described (*37*). The instrument was run in high-resolution mode (mass resolution of ~700) with internally calibrated dual-count detection. Noise reduction and cell extraction parameters were as follows: cell length, 10 to 65; lower convolution threshold, 10. Samples were normalized using beta beads (*64*).

### X-shift analysis and graphic display of single-cell mass cytometry data

Pooled aRMS and pooled eRMS cells were clustered on the basis of a combination of surface markers, myogenic transcription factors, and cell cycle markers (CD44, Axl, Pax-7, myogenin, IdU) using the X-shift algorithm. To visualize the spatial relationships between cells within these X-shift clusters, 2000 randomly sampled cells from each cluster were subjected to a force-directed layout (*38*). All conditions were processed simultaneously so that the resulting map would capture all populations present in the entire dataset.

### RMS patient gene expression

To measure myogenic gene expression in RMS patients, we analyzed a published RMS patient gene expression dataset (*35*) available on the R2: Genomics Analysis and Visualization Platform (http://r2.amc.nl). We computed expression of *PAX7*, *MYOD1*, *MYOG*, and *MYH8* genes subsetting across aRMS or eRMS patients.

### Immunofluorescence

To assess Pax-7, myogenin, or MyHC positivity, cells were first washed with PBS and then fixed with ROTI Histofix 4 % (Carl Roth, P087.3) for 30 min. Following three 5-min washes with PBS using an automated plate washer (BioTek 50 TS washer, Agilent Technologies), cells were permeabilized with 0.1% Triton X-100 (Sigma-Aldrich, X100) in PBS for 15 min. After three 5-min washes with PBS and blocking with 1% BSA (Sigma-Aldrich, A8022) in PBS for 1 hour, cells were incubated for 2 hours at room temperature or overnight at 4°C with primary antibody diluted in 1% BSA. Cells were washed three times for 5 min with PBS and incubated with secondary antibodies diluted 1:300 in 1% BSA. Last, cells were washed again three times for 5 min with PBS and covered with 1:1000 solution of Hoechst 33342 (Thermo Fisher Scientific, 62249) in PBS. Images were acquired and quantified with an automated workflow on PerkinElmer Operetta. Primary antibodies used include myogenin (M-225: Santa Cruz Biotechnology, sc-576, or Thermo Fisher Scientific, PA5-78067; dilution: 1:300), MyHC [MF-20: Developmental Studies Hybridoma Bank (DSHB); dilution: 1:500], and Ki-67 (Thermo Fisher Scientific, 14-5698-82, or Dako, M7240; dilution: 1:1000). Secondary antibodies used included Goat anti-Mouse IgG (H+L) Highly Cross-Adsorbed Secondary Antibody Alexa Fluor 594 (Thermo Fisher Scientific, A-11032), Goat anti-Rabbit IgG (H+L) Highly Cross-Adsorbed Secondary Antibody Alexa Fluor 488 (Thermo Fisher Scientific, A-11034), and Goat Anti-Rat IgG (H+L) Highly Cross-Adsorbed Secondary Antibody Alexa Fluor 647 (Thermo Fisher Scientific, A21247) at a dilution of 1:1000.

### Immunohistochemistry

PDX tumor pieces were first fixed with ROTI Histofix 4% (Carl Roth, P087.3), embedded with paraffin as Formalin-Fixed Paraffin-Embedded (FFPE) tissues, and cut in sections of 2 μM before staining on the BOND Fully Automated Immunohistochemistry Staining System (Leica). The sections were incubated for 30 min with primary antibodies against PAX7 (DSHB; dilution: 1:100), myogenin (Cell Marque Lifescreen, 296 M-14; dilution: 1:20), MyHC (DSHB; dilution: 1:500), and Ki-67 (Cell Marque Lifescreen, 275R-16; dilution: 1:100). Visualization of the antibodies was performed with a Bond refine detection system (Leica). All sections were counterstained with hematoxylin.

### Western blotting

Whole-cell lysates were extracted using radioimmunoprecipitation assay lysis buffer [50 mM tris-HCl (pH 7.5), 150 mM NaCl, 1% NP-40, 0.5% Na-deoxycholate, 0.1% SDS, 1 mM EGTA, 50 mM NaF, 5 mM Na_4_P_2_O_7_, 1 mM Na_3_VO_4_, and 10 mM ß-glycerol phosphate] in the presence of the cOmplete, Mini, EDTA-free Protease Inhibitor Cocktail (Sigma-Aldrich, 11836170001). Protein concentration was measured with the Pierce BCA Protein Assay Kit (Thermo Fisher Scientific, 23227) according to the manufacturer’s instructions. Whole-cell lysates (5 to 20 μg) were reduced with Laemmli sample buffer (Bio-Rad, 161-0747) supplemented with 1:20 dithiothreitol. After boiling the samples at 95°C for 5 min, proteins were separated using NuPAGE 4 to 12%, bis-tris, 1.0-mm, Mini Protein Gels (Thermo Fisher Scientific, NP0323BOX). Gels were transferred on membranes using the Trans-Blot Turbo Transfer System (Bio-Rad, 1704150). Following blocking with 5% milk (Carl Roth, T145.3) in tris-buffered saline (TBS)/0.05% Tween (Sigma-Aldrich, P9416) for 30 to 60 min, membranes were incubated overnight at 4°C or for 2 hours at room temperature with primary antibodies diluted 1:1000. After three washing steps with TBS/0.05% Tween for 5 min, membranes were incubated for 1 hour at room temperature with horseradish peroxidase (HRP)–linked secondary antibodies diluted 1:5000. Last, after three additional washing steps with TBS/0.05% Tween for 5 min, proteins were detected by chemiluminescence using SuperSignal West Femto Maximum Sensitivity Substrate (Thermo Fisher Scientific, 34095) and a ChemiDoc MP imager (Bio-Rad). The following antibodies were used: Pax-7 (DSHB), MyoD (M-318, Santa Cruz Biotechnology, sc-760), myogenin (M-225, Santa Cruz Biotechnology, sc-576, or Thermo Fisher Scientific, PA5-78067), PAX3::FOXO1 (FKHR H-128, Santa Cruz Biotechnology, sc-11350), MyHC (MF-20, DSHB), glyceraldehyde-3-phosphate dehydrogenase (GAPDH; Cell Signaling Technology, 5174S), phosphorylated ERK (Cell Signaling Technology, 9101S), ERK1/2 (Cell Signaling Technology, 9102 L), anti-rabbit IgG HRP-linked antibody (Cell Signaling Technology, 7074S), and anti-mouse IgG HRP-linked antibody (Cell Signaling Technology, 7076S).

### Real-time qRT-PCR

Total RNA was extracted using the RNeasy Micro Kit (Qiagen, 74004) from cultured cells and using the RNeasy Mini Kit (Qiagen, 74106) from tumor pieces according to the manufacturer’s instructions. Complementary DNA (cDNA) was synthesized using the High-Capacity cDNA Reverse Transcription Kit with ribonuclease (RNase) inhibitor (Thermo Fisher Scientific, 4374967) modifying the total volume to 10 μl. Specifically, for each sample, a mix consisting of 1.0 μl of 10X RT Buffer, 0.4 μl of 25XdNTPs Mix (100 mM), 1.0 μl of 10X RT Random Primers, 0.5 μl of MultiScribe Reverse Transcriptase, 0.5 μl of RNase inhibitor, 1.6 μl of Nuclease-free H_2_O, and 5 μl of Nuclease-Free Water (Thermo Fisher Scientific, AM9937) was incubated in a thermocycler according to the manufacturer’s instructions. Each sample was analyzed by qRT-PCR using TaqMan Gene Expression Master Mix (Thermo Fisher Scientific, 4369542) and TaqMan gene expression assays. qRT-PCR was performed in technical triplicate for each sample on a 7900HT Fast Real-Time PCR machine. To calculate the relative gene expression of each gene, the 2^−∆∆Ct^ method was used and the quantity of RNA was normalized to the internal control GAPDH. The following TaqMan gene expression assays were used: CD44 (Hs01075864_m1), AXL (Hs01064444_m1), PAX7 (Hs00242962_m1), MYOD1 (Hs00159528_m1), MYOG (Hs01072232_m1), MYH3 (Hs01074230_m1), and GAPDH (Hs02758991_g1).

### Flow cytometry and sorting

CD44^+^ and CD44^−^ subpopulations were sorted on BD FACSAria Fusion using fluorescein isothiocyanate (FITC) anti-human CD44 antibody (BioLegend, 338804) at 1:200 dilution. CD44-positive and CD44-negative cells were gated on the basis of the FITC mouse IgG1 (BioLegend, 400110) isotype control. To exclude dead cells from sorting, cells were stained with eBioscience 7-AAD Viability Staining Solution (Thermo Fisher Scientific, 00-6993-50) before sorting. For stability experiments, 50,000 cells per well from CD44^+^, CD44^−^, or unsorted populations were cultured in 24-well plates and analyzed on LSRFortessa (BD) at regular intervals for 3 weeks after fresh staining with FITC anti-human CD44 antibody (BioLegend, 338804) as described above.

To measure AXL, CD44, and CD105 expression after drug treatment, cells were treated with drugs for 48 hours as described below and detached from the plates for staining. Cells were first stained with the Zombie NIR Fixable Viability Kit (BioLegend, 423105) according to the manufacturer’s instructions to gate out dead cells. Flow cytometry was performed by labeling cells for 30 min at 4°C with the following antibodies (dilution of 1:50): FITC anti-human CD44 (BioLegend, 338804), phycoerythrin (PE) anti-CD105 (BioLegend, 800503), and allophycocyanin (APC) Axl (Thermo Fisher Scientific, 17-1087-41). Isotype controls were used to gate positive/negative cells and included the following antibodies: FITC mouse IgG1 (BioLegend, 400110), APC mouse IgG1 (BioLegend, 400122), and PE mouse IgG1 (BioLegend, 400114).

For Ki-67 staining of CD44^+^ and CD44^−^ subpopulations, cells were first stained with FITC anti-human CD44 antibody (BioLegend, 338804) or with the corresponding isotype control, followed by Zombie NIR Fixable Viability Kit (BioLegend, 423105) staining as described above. Cells were then fixed in 2% PFA using ROTI Histofix (Carl Roth, P087.3) and stained with PE Mouse Anti-Ki-67 Set (BioLegend, 556027) according to the manufacturer’s instructions.

### Drug treatment

For WB, qRT-PCR, and cell cycle analysis, cells were plated in six-well plates at a concentration of 300,000 cells per well, equilibrated overnight, and then treated with drugs for the indicated time. In case of trametinib, cells were treated immediately on the day of plating. For WB analysis of phosphorylated ERK, cells were plated in six-well plates at a concentration of 300,000 cells per well, equilibrated overnight, and then treated with 5 or 10 nM trametinib for 3 hours.

For flow cytometry analysis, cells were plated in 24-well plates at a concentration of 100,000 cells per well, equilibrated overnight, and then treated with chemotherapy for 48 hours.

For immunofluorescence, cells were plated in 384-well plates at a concentration of 2000 to 4000 cells per well, equilibrated overnight, and then treated with the indicated drugs for 72 hours.

To test the drug sensitivity of CD44^+^ and CD44^−^ FACS-sorted subpopulations, cells were sorted as described above. After sorting, CD44^+^, CD44^−^, and an additional unsorted reference population were plated in 384-well plates coated with Matrigel at a cell density of 800 cells per well. The day after, the medium was replaced, and cells were incubated for further 72 hours with the indicated drugs. Data of each CD44^+^, CD44^−^, and unsorted populations were normalized to DMSO (vehicle)–treated conditions, defined as 100% viability. IC_50_ values were determined from the dose-response curves generated using GraphPad Prism. Drugs included vincristine sulfate (ApexBio, A1765-5.1), 4-HC (Niomech, CAS 39800-16-3), etoposide (Selleckchem, S1225), trametinib (Selleckchem, S2673), dabrafenib (Selleckchem, S2807), and regorafenib (Selleckchem, S1178).

### High-content single-cell imaging drug screening with MYOscopy

aRMS-1 (IC-pPDX-104) and aRMS-3 (IC-pPDX-35) cells were plated in 384-well plates coated with Matrigel at a cell density of 2000 to 4000 cells per well. The day after, cells were treated with a drug library containing 244 drugs at a concentration of 10, 1, 0.1, or 0.01 μM. After 72 hours of drug incubation, cells were processed for MYOscopy to determine their cellular composition based on markers identified by single-cell analysis. To do so, cells were processed for immunofluorescence as described above and stained with the following primary antibodies: myogenin (M-225, Santa Cruz Biotechnology, sc-576, or Thermo Fisher Scientific, PA5-78067; dilution: 1:300), MyHC (MF-20, DSHB; dilution: 1:500), and Ki-67 (Thermo Fisher Scientific, 14-5698-82; dilution: 1:1000). Secondary antibodies used included Goat anti-Mouse IgG (H+L) Highly Cross-Adsorbed Secondary Antibody Alexa Fluor 594 (Thermo Fisher Scientific, A-11032), Goat anti-Rabbit IgG (H+L) Highly Cross-Adsorbed Secondary Antibody Alexa Fluor 488 (Thermo Fisher Scientific, A-11034), and Goat anti-Rat IgG (H+L) Highly Cross-Adsorbed Secondary Antibody Alexa Fluor 647 (Thermo Fisher Scientific, A21247) at a dilution of 1:1000. Cells were assigned to the corresponding myogenic states according to expression of the following markers: quiescent MuSC-like (myogenin^−^Ki-67^−^), cycling MuSC-like (myogenin^−^Ki-67^+^), cycling progenitors (myogenin^+^Ki-67^+^MyHC^−^), noncycling committed progenitors (myogenin^+^Ki-67^−^MyHC^−^), and differentiated (MyHC^+^). An unstained control was included on every plate and used as a reference for signal background. Differentiating hits were defined as drugs increasing the percentage of noncycling committed progenitors and differentiated cells, and decreasing cycling progenitors compared to untreated controls; dedifferentiating hits were defined as drugs increasing the percentage of MuSC-like cells compared to untreated controls of at least 1.5-fold. An overall “differentiating score” was calculated as follows: [% noncycling committed progenitors (myogenin^+^Ki-67^−^MyHC^−^) + % differentiated (MyHC^+^) − % cycling progenitors (myogenin^+^Ki-67^+^MyHC^−^)]_compound, 1 μM_ − [% committed (myogenin^+^Ki-67^−^MyHC^−^) + % differentiated (MyHC^+^) − % cycling progenitors (myogenin^+^Ki-67^+^MyHC^−^)_untreated control_, whereas an overall “de-differentiating score” was calculated as follows: [% quiescent MuSC-like (myogenin^−^Ki-67^−^) + % cycling MuSC-like (myogenin^−^Ki-67^+^)]_compound, 1 μM_ − [% quiescent MuSC-like (myogenin^−^Ki-67^−^) + % cycling MuSC-like (myogenin^−^Ki-67^+^)]_untreated control_.

For the trametinib combination screening, aRMS-1 (IC-pPDX-104) cells were plated in 384-well plates coated with Matrigel at a cell density of 4000 cells per well. The day after, cells were treated with the drug library containing 244 drugs at a concentration of 10, 1, 0.1, or 0.01 μM and with additional 50 nM trametinib (Selleckchem, S2673). As a control, untreated cells were included on every plate. After 72 hours of incubation, cells were processed for MYOscopy as described above. The “combined differentiating score” was calculated averaging the “differentiating scores” calculated at 10 μM, 1 μM, 100 nM, and 10 nM and subtracting the differentiating score of 50 nM trametinib alone.

### Cell cycle analysis

After treatment with trametinib as described above, cells were detached from the plates, washed in PBS, fixed in ice-cold 70% ethanol, and incubated at −20°C for >2 hours. Cells were then washed with PBS and resuspended in 500 μl of propidium iodide (PI) solution [PI (0.4 mg/ml) and RNase A (0.4 μg/ml) in PBS with 0.0001% Triton X-100] before analysis on the LSRFortessa (BD) Cell Analyzer. All FACS analyses were done on FlowJo v10.8 software.

### Quantification and statistical analysis

Data throughout the article are presented as individual values with SEM. Statistical analyses were performed using GraphPad Prism v9.0 (GraphPad Software) or within the Seurat R package (for scRNAseq data). Significance of mean differences was performed using statistical details listed in the figure legends. All FACS analyses were analyzed on FlowJo v10.8 software.

To assess the clinical value of the aRMS subpopulations identified by scRNAseq, we first generated cluster-associated gene signatures by pairwise comparisons between all the clusters identified by scRNAseq in the combined aRMS primary culture dataset. Subpopulation-specific markers were identified using the likelihood ratio test (*65*) implemented in the FindAllMarkers function in Seurat and defined as genes overexpressed with a log fold change of >0.25 and *P* < 0.05 following Bonferroni correction. Next, we analyzed a previously published RMS patient gene expression dataset (*35*) using the R2: Genomics Analysis and Visualization Platform (http://r2.amc.nl). For each gene signature, we computed the average log fold change in expression between living (status = live) and deceased (status = dead) patients, where the averaging is over all the genes in the signature. Here, we consider both up-regulated and down-regulated genes. However, the down-regulated genes contribute to the average log fold change with minus signs. To obtain a null distribution for this test statistic, we randomly flip the “survivor” and “nonsurvivor” labels when computing average log fold change. We simulate this null distribution to compute the *P* value for each gene signature.

## Supplementary Materials

### This PDF file includes:

Figs. S1 to S7 References
