# Supplementary material for: Single-cell profiling of alveolar rhabdomyosarcoma reveals RAS pathway inhibitors as cell-fate hijackers with therapeutic relevance
Source: Sci Adv. 2023 Feb 8;9(6):eade9238. doi: 10.1126/sciadv.ade9238 (PMC9908029; doi:10.1126/sciadv.ade9238)
Supplement: Supplementary file 1 — Figs. S1 to S7 References [file sciadv.ade9238_sm.pdf]

Science Advances
AAAS

Supplementary Materials for

Single-cell profiling of alveolar rhabdomyosarcoma reveals RAS pathway inhibitors as cell-fate hijackers with therapeutic relevance

Sara G. Danielli *et al.*

Corresponding author: Beat W. Schäfer, Beat.Schaefer@kispi.uzh.ch; Marco Wachtel, Marco.Wachtel@kispi.uzh.ch; Ermelinda Porpiglia, eporpiglia@biomed.au.dk

Sci. Adv. **9**, eade9238 (2023)
DOI: 10.1126/sciadv.ade9238

The PDF file includes:

Figs. S1 to S7
Legends for tables S1 to S10
References

Other Supplementary Material for this manuscript includes the following:

Tables S1 to S10

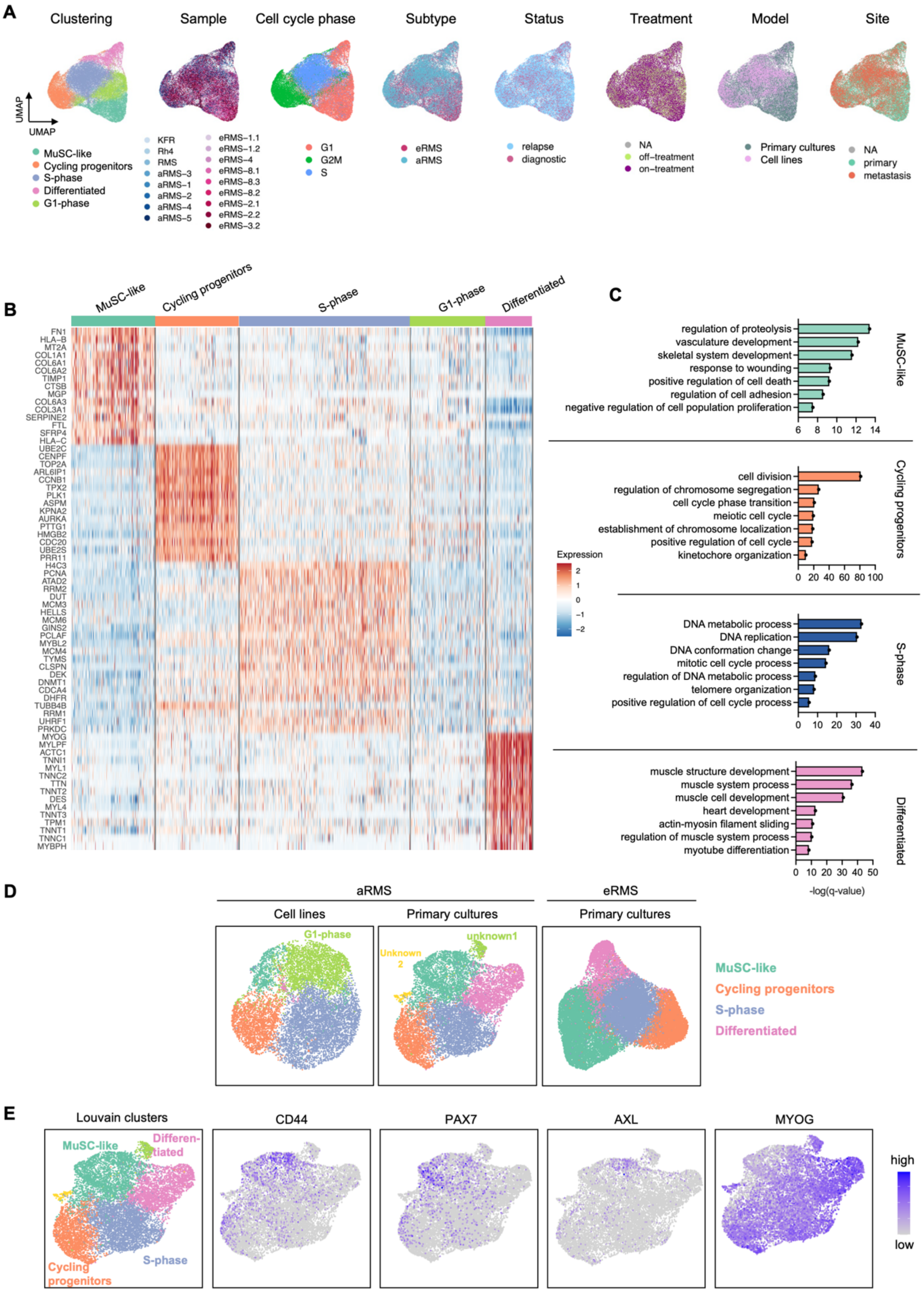

Fig. S1: RMS single-cell gene signatures.

A. UMAP plot of 48,859 RMS cells after integration. Cells are colored based on the populations identified by Louvain clustering, the sample of origin, the inferred cell cycle status, the RMS subtype, the status, treatment and site of the tumor at the time of PDX generation and on the model. **B.** Heatmap of the top 15 gene markers for each cluster (top bars) across the combined RMS dataset. Relative $\log_2$ fold change of gene expression (color bar) is shown **C.** Top seven biological processes enriched in each cluster calculated by Metascape (*30*). **D.** UMAP plots of combined aRMS primary cultures or cell lines and of eRMS primary cultures after removing inter-sample differences by SCT algorithm. The identified cellular states are indicated. **E.** UMAP plots of combined aRMS primary cultures colored based on the identified Louvain clusters (left panel) or based on expression of CD44, PAX7, AXL and MYOG.

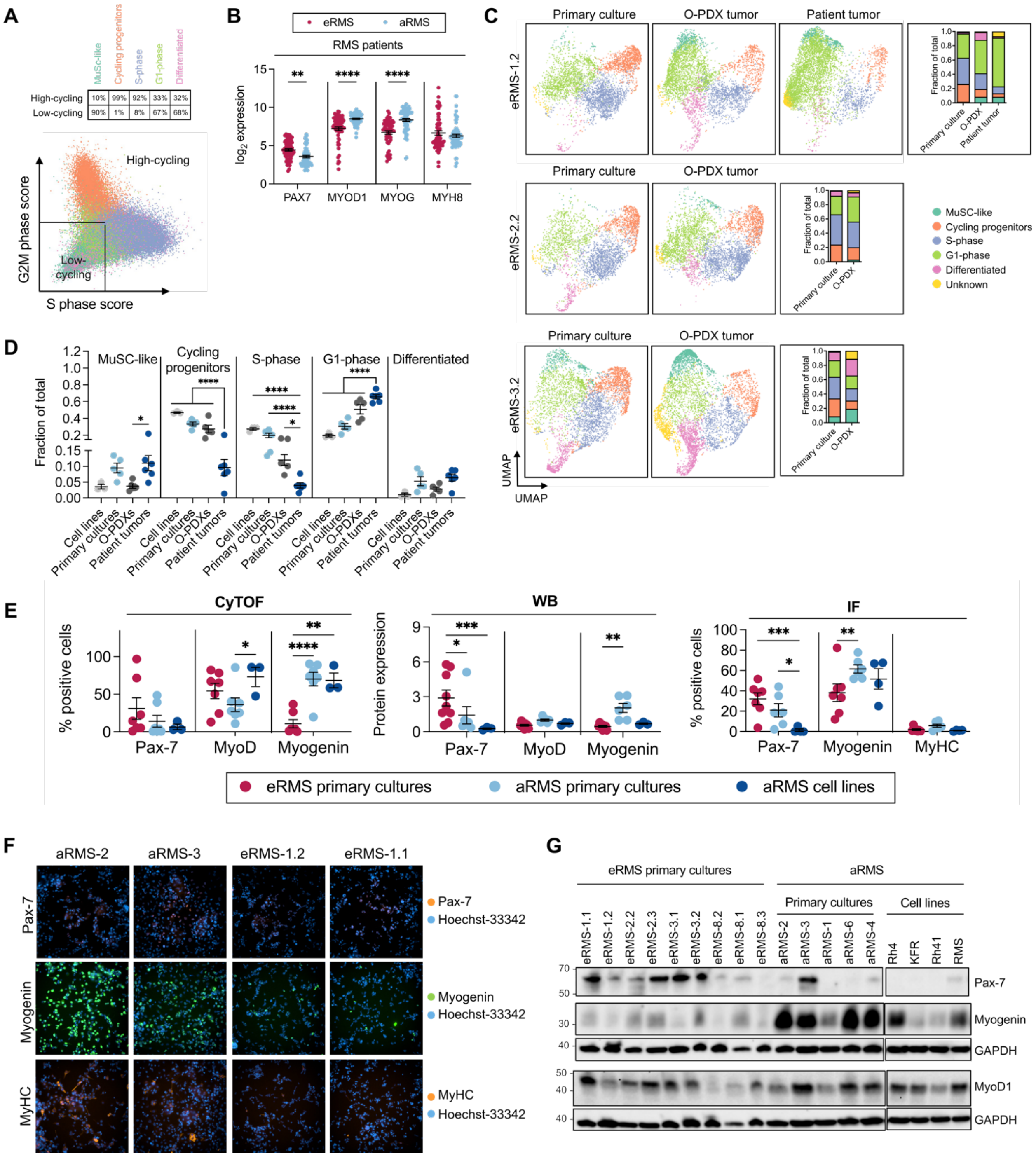

Fig. S2: scRNAseq characterization of RMS models and subtypes.

A. Proportion of high-cycling (cells in G2M or S phase) and low-cycling RMS cells (cells in G1 phase) across the identified Louvain clusters. Cells are considered high-cycling if S-phase or G2M scores >0 and low-cycling if S-phase and G2M-phase scores <0. **B.** Transcript levels of depicted

markers in patient's samples (*n* = 65 aRMS; *n* = 60 eRMS, r2.aml.nl). Individual data points and mean ± SEM are shown; ordinary two-way ANOVA with uncorrected Fisher's LSD. **C.** Single-cell transcriptomic comparison of three eRMS primary cultures and of their originating O-PDXs and/or patient tumor (*36*). UMAP plots colored by the identified Louvain clusters, and their relative distribution across samples is shown. **D.** Relative proportion of Louvain clusters across different aRMS preclinical models and patient tumors (*36*). Data are represented as mean ± SEM of *n* = 6 patient tumors, *n* = 5 O-PDXs, *n* = 5 primary cultures and *n* = 3 cell lines; ordinary two-way ANOVA with uncorrected Fisher's LSD. **E.** Percentage of positive cells and expression levels of markers delineating a myogenic progression (Pax-7, *MuSC-like cells*; MyoD, *activated progenitor cells*; myogenin, *committed progenitors/differentiated* cells; MyHC, *differentiated* cells) across individual RMS primary cultures and cell lines measured by CyTOF, western blot (protein expression was normalized to GAPDH) and immunofluorescence. Data are represented as mean ± SEM of the indicated number of samples; ordinary two-way ANOVA with uncorrected Fisher's LSD. **F.** Representative immunofluorescence images of aRMS and eRMS primary cultures stained for Pax-7, myogenin or MyHC. **G.** Representative western blots of Pax-7, MyoD and myogenin protein expression in a panel of RMS primary cultures and cell lines. GAPDH was used as a loading control.

*, $P < 0.05$; **, $P < 0.01$; ***, $P < 0.001$; ****, $P \leq 0.0001$.

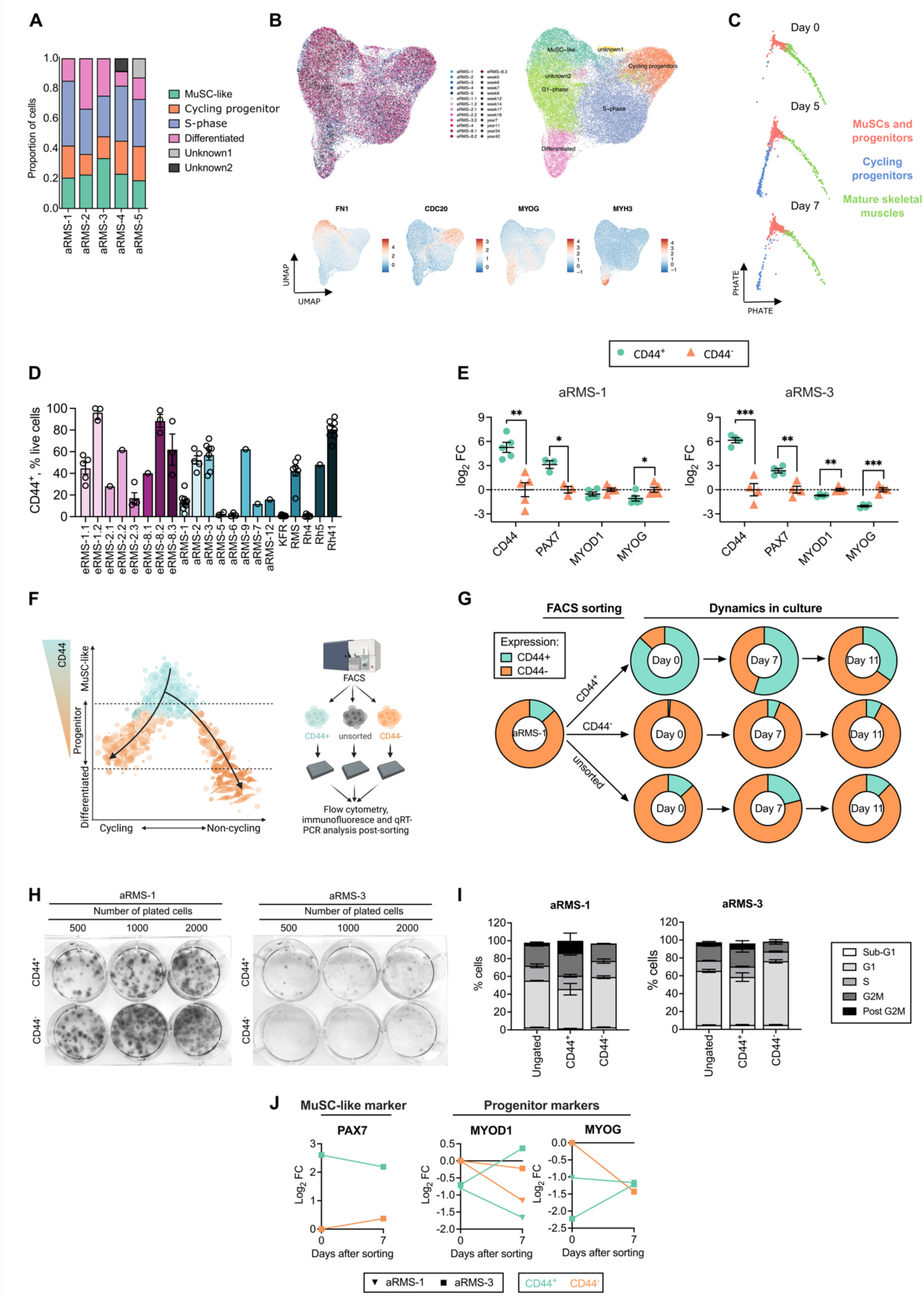

Fig. S3. Hierarchical organization of RMS tumors.

A. Fraction of cells belonging to the identified clusters across *n* = 5 aRMS primary cultures after integration using SCT-correction. Only clusters that were commonly shared across different cultures ("MuSC-like", "Cycling progenitor", "S-phase", "Differentiated") were selected for pseudotime analysis. The clusters "Unknown1" and "Unknown2" were only found in the sample aRMS-5, respectively aRMS-4. For this reason, both clusters were excluded for PHATE dimensionality reduction and pseudotime trajectory calculation. **B.** Integrated UMAP plots of RMS primary cultures and developing human skeletal muscles (*42*). Cells are colored by the sample of origin (top left panel), identified Louvain clusters (top right panel), or by expression of markers delineating a myogenic progression (lower panel). **C.** PHATE plots of mouse muscle scRNAseq dataset (*32*) at different days (0, 5, 7) after muscle injury. Cells are colored based on the subpopulations identified in the original publication. **D.** CD44 cell surface expression measured by flow cytometry in a panel of primary cultures and cell lines of eRMS (pink) and aRMS (blue) subtypes. Data are represented as mean ± SEM of the indicated number of biological replicates. **E.** qRT-PCR data generated from FACS-sorted $CD44^+$ and $CD44^-$ subpopulations. Log_2 fold change mRNA levels of $CD44^+$ normalized to $CD44^-$ are depicted. Data are represented as mean ± SEM of $n \geq 4$ biological replicates; multiple unpaired *t*-tests. **F.** FACS workflow. **G.** Flow cytometry analysis of the stability of $CD44^+$ and $CD44^-$ subpopulations in aRMS-1 (IC-pPDX-104) cells. Unsorted reference is also shown. Data are represented as mean ± SEM of $n \geq 2$ biological replicates. **H.** Colony-forming ability of FACS-sorted $CD44^+$ and $CD44^-$ subpopulations from aRMS-1 and aRMS-3 cells stained with crystal violet 14 or 21 days, respectively, after plating. **I.** Cell cycle distribution of $CD44^+$, $CD44^-$ and ungated subpopulations. Data are represented as mean ± SEM of $n \geq 2$ biological replicates. **J.** Time-course qRT-PCR analysis of sorted $CD44^+$ and $CD44^-$ subpopulations of three primary aRMS cultures. Log_2 fold

change (FC) to the $CD44^-$ subpopulation at time point 0 is shown. aRMS-1 (IC-pPDX-104) cells express no PAX7, therefore there is no data point.

*, $P < 0.05$; **, $P < 0.01$; ***, $P < 0.001$

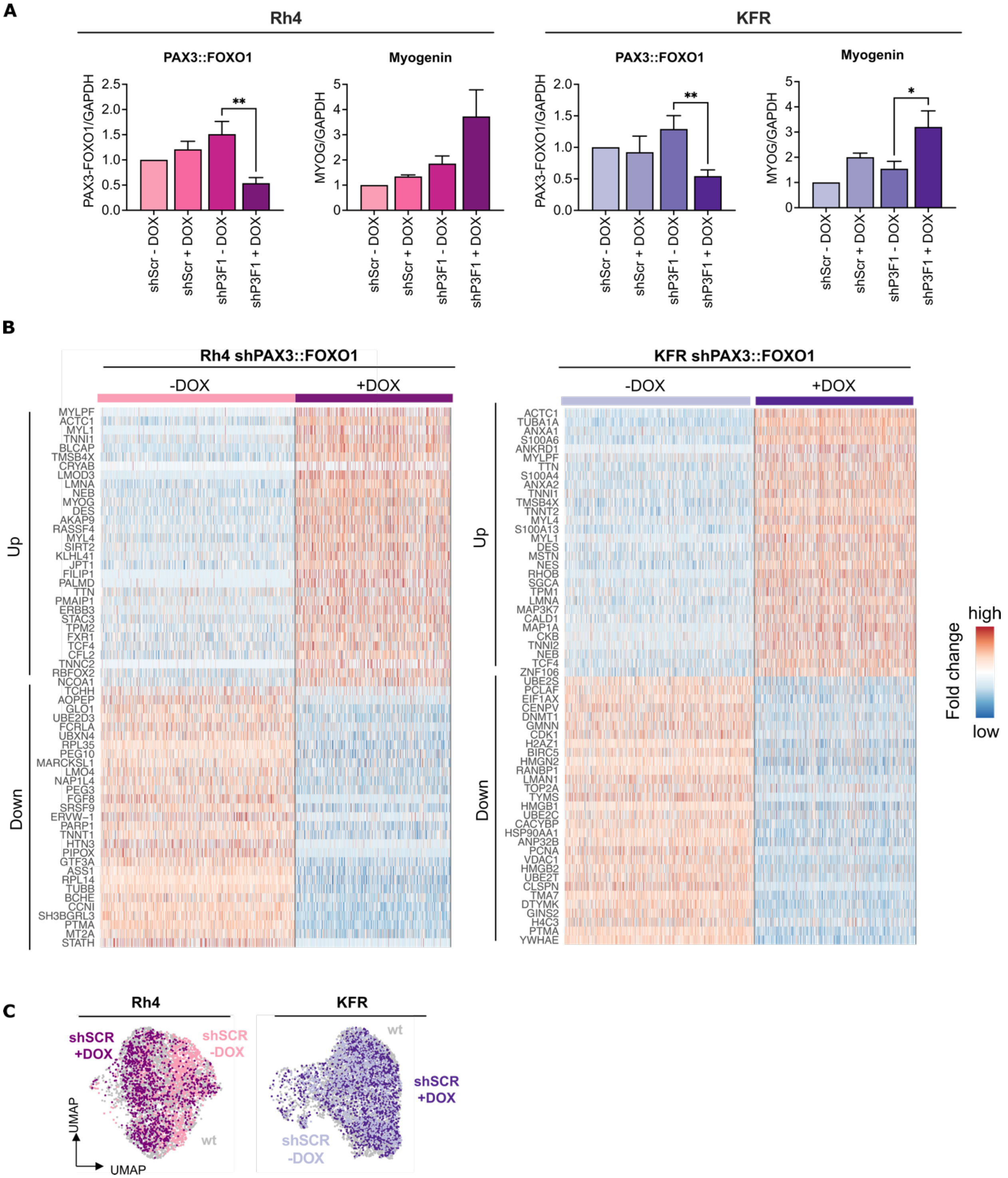

Fig. S4. Single-cell responses upon PAX3::FOXO1 downregulation.

A. Western blot quantification of Rh4 or KFR cells treated with or without doxycycline (DOX) for 48 hrs. Data are represented as mean ± SEM of $n \geq 5$ biological replicates; ordinary one-way

ANOVA with Dunnet's multiple comparison test. *, $P < 0.05$; **, $P < 0.01$. **B.** Heatmap plots showing $\log_2$ fold change of the genes differentially expressed ($\log_2$ fold change > 0.5) in Rh4 (left) or KFR (right) cells transduced with shPAX3::FOXO1 construct upon doxycycline (DOX) treatment. Relative $\log_2$ fold change of gene expression (color bar) is shown. **C.** UMAP plot of shSCR and *wt* Rh4 and KFR cells used as a control for PAX3::FOXO1 downregulation.

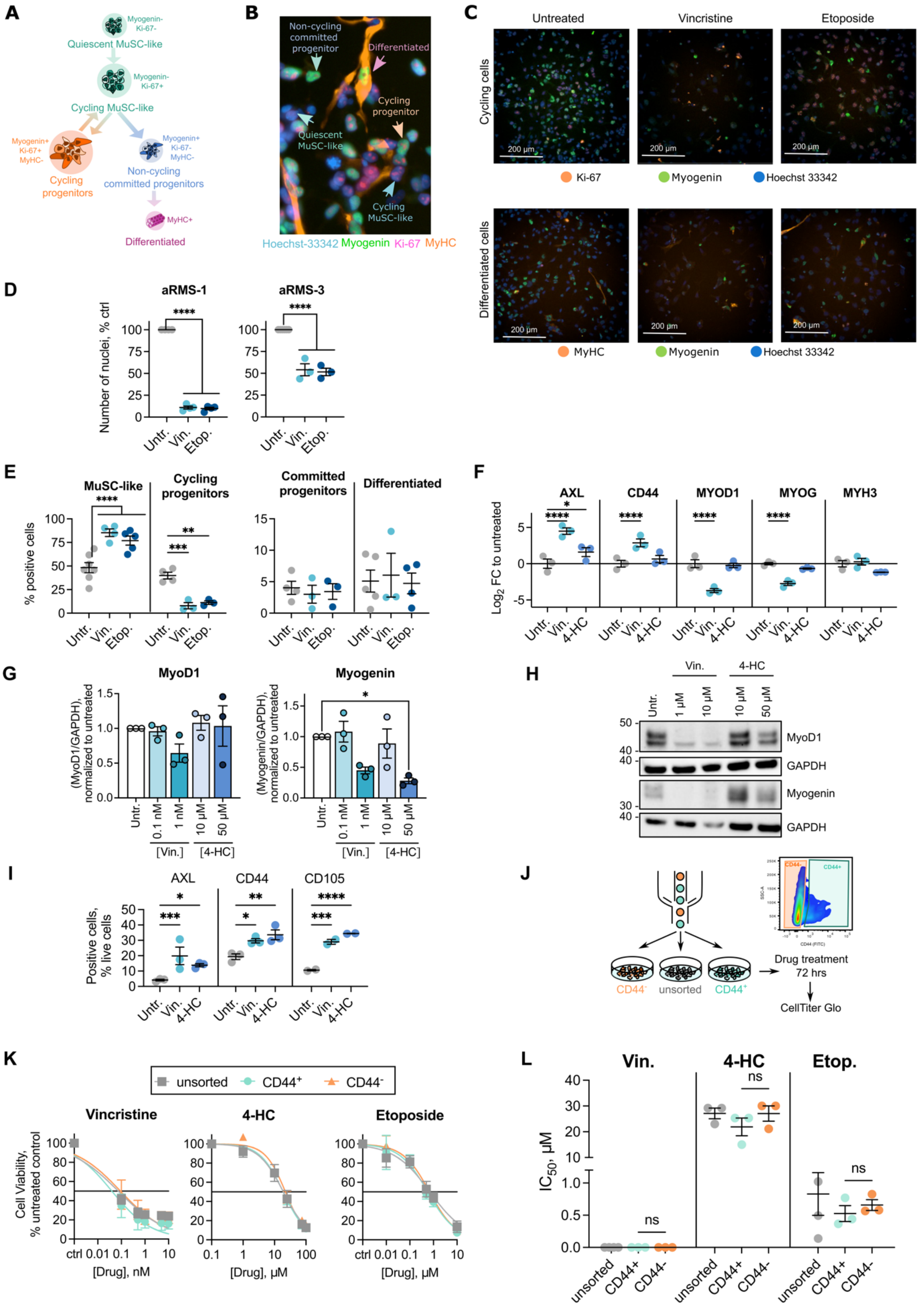

Fig. S5: Effect of chemotherapy on aRMS cellular composition.

A. Myogenin, Ki-67 and MyHC marker positivity across the aRMS cellular states identified by single-cell analysis. **B.** Representative immunofluorescence analysis of aRMS-1 (IC-pPDX-104) cells. **C.** Representative images of aRMS-3 (IC-pPDX-35) cells exposed to 1 μM vincristine sulfate or to 10 μM etoposide for 72 hrs. **D.** Number of nuclei remaining after a 72 hrs exposure to vincristine sulfate [10 nM in aRMS-1 (IC-pPDX-104), 10 μM in aRMS-3 (IC-pPDX-35) cells] or etoposide [1 μM in aRMS-1 (IC-pPDX-104), 10 μM in aRMS-3 (IC-pPDX-35) cells]. Data are represented as mean ± SEM of $n \geq 3$ biological replicates; ordinary one-way ANOVA with Dunnett's multiple comparison test. **E.** Immunofluorescence quantification of aRMS-3 (IC-pPDX-35) cells exposed to 10 μM vincristine sulfate or 10 μM etoposide for 72 hrs. Data are represented as mean ± SEM of $n \geq 3$ biological replicates; ordinary two-way ANOVA with uncorrected Fisher's LSD. **F.** qRT-PCR data generated with aRMS-3 cells (IC-pPDX-35) exposed to 10 μM vincristine sulfate or 50 μM 4-HC for 48 hrs. Data are represented as mean ± SEM of $n = 3$ biological replicates; ordinary two-way ANOVA with uncorrected Fisher's LSD. **G.** Western blot quantification of aRMS-1 (IC-pPDX-104) cells exposed to vincristine or to 4-HC for 48 hrs at the indicated concentrations. Data are represented as mean ± SEM of $n = 3$ biological replicates; ordinary two-way ANOVA with Dunnett's multiple comparison test. **H.** Representative western blot of aRMS-3 (IC-pPDX-35) cells exposed to vincristine or to 4-HC for 48 hrs at the indicated concentrations. **I.** Quantification of FACS analysis of aRMS-1 (IC-pPDX-104) cells treated with 1 nM vincristine or 10 μM 4-HC for 48 hrs. Data are represented as mean ± SEM of $n = 3$ biological replicates ($n = 2$ for CD105); ordinary two-way ANOVA with uncorrected Fisher's LSD. **J.** Experimental workflow. aRMS-1 (IC-pPDX-104) cells were first FACS-sorted based on expression of CD44, then exposed to the indicated drugs. After 72 hrs, viable cells were quantified by CellTiter Glo. Unsorted cells were used as a reference. **K.** Dose-response curves of vincristine

sulfate, 4-HC and etoposide in FACS-sorted aRMS-1 (IC-pPDX-104) subpopulations. Data are represented as mean ± SEM of $n \geq 3$ biological replicates; **L.** Half maximal inhibitory concentration (IC_{50}) values of vincristine, 4-HC and etoposide in aRMS-1 (IC-pPDX-104) cells. Data are represented as mean ± SEM of $n \geq 3$ biological replicates.

Untr.: untreated; *Vin.*: vincristine sulfate; *Etop.*: etoposide; *4-HC*: 4-hydroperoxycyclophosphamide. *, $P < 0.05$; **, $P < 0.01$; ***, $P < 0.001$.; ****, $P < 0.0001$.

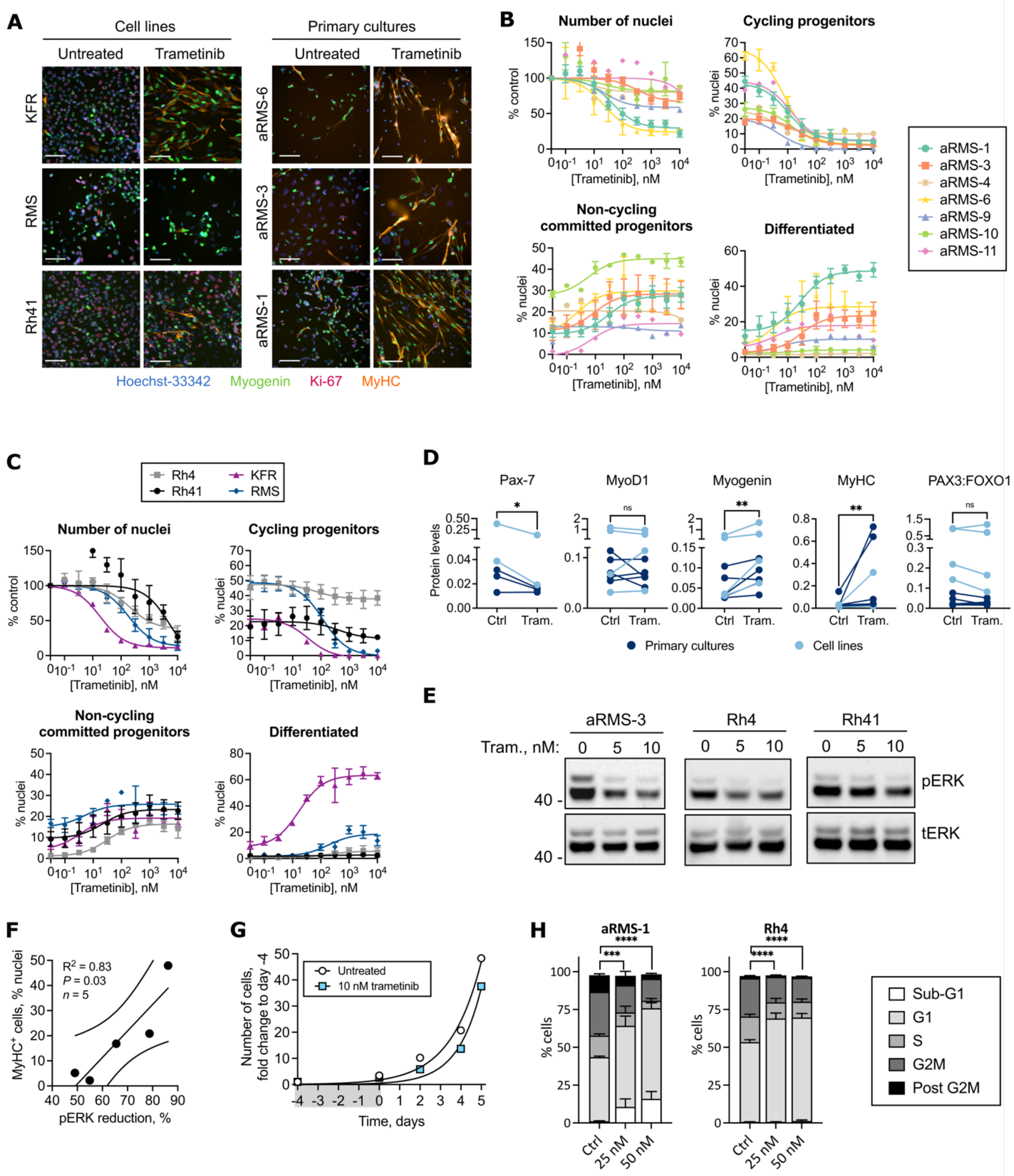

Fig. S6: Trametinib effect on aRMS primary cultures and cell lines.

A. Representative images of aRMS primary cultures and cell lines exposed to 100 nM trametinib for 72 hrs. Scale bar = 100 μm. **B and C.** Dose-response curves of aRMS primary cultures (**B**) and cell lines (**C**) exposed to trametinib for 72 hrs. The number of nuclei, percentage of cycling, non-

cycling committed progenitors and differentiated cells was measured with MYOscopy. Data are represented as mean of n = 1-6 biological replicates for each sample. **D.** Western blot quantification of changes in protein levels following treatment with 50 nM trametinib for 96 hrs in aRMS primary cultures and cell lines. Protein levels were normalized to GAPDH. **E.** Representative western blots of phosphorylation of ERK (pERK) in the primary culture aRMS-3 (IC-pPDX-35) and in the cell lines Rh41 and Rh4 after exposure to 5 or 10 nM trametinib for 3 hrs. The protein levels of total ERK (tERK) are also shown. **F.** Correlation between the reduction in ERK phosphorylation after 3 hrs exposure to 10 nM Trametinib and the percentage of differentiated cells (MyHC+) after exposure to 1 μM Trametinib for 72 hrs measured by MYOscopy. Data points, representing different aRMS samples, are interpolated with a linear regression. Correlation coefficient (R^2), statistical significance (P) and number of data points (n) are indicated. **G.** Proliferation curve of aRMS-1 cells exposed to trametinib for 4 days (grey bar) and cultivated in drug-free medium for further 5 days, as determined by cell counting. **H.** Quantification of cell cycle phases after exposure of aRMS-1 (IC-pPDX-104) or Rh4 cells to the indicated concentrations of trametinib or to vehicle controls for 96 hrs. Data are represented as mean ± SEM of $n \geq 3$ biological replicates; ordinary two-way ANOVA with Dunnet's multiple comparison test.

Ctrl., untreated control; Tram., trametinib.

*, $P < 0.05$; **, $P < 0.01$; ***, $P < 0.001$.; ****, $P < 0.0001$.

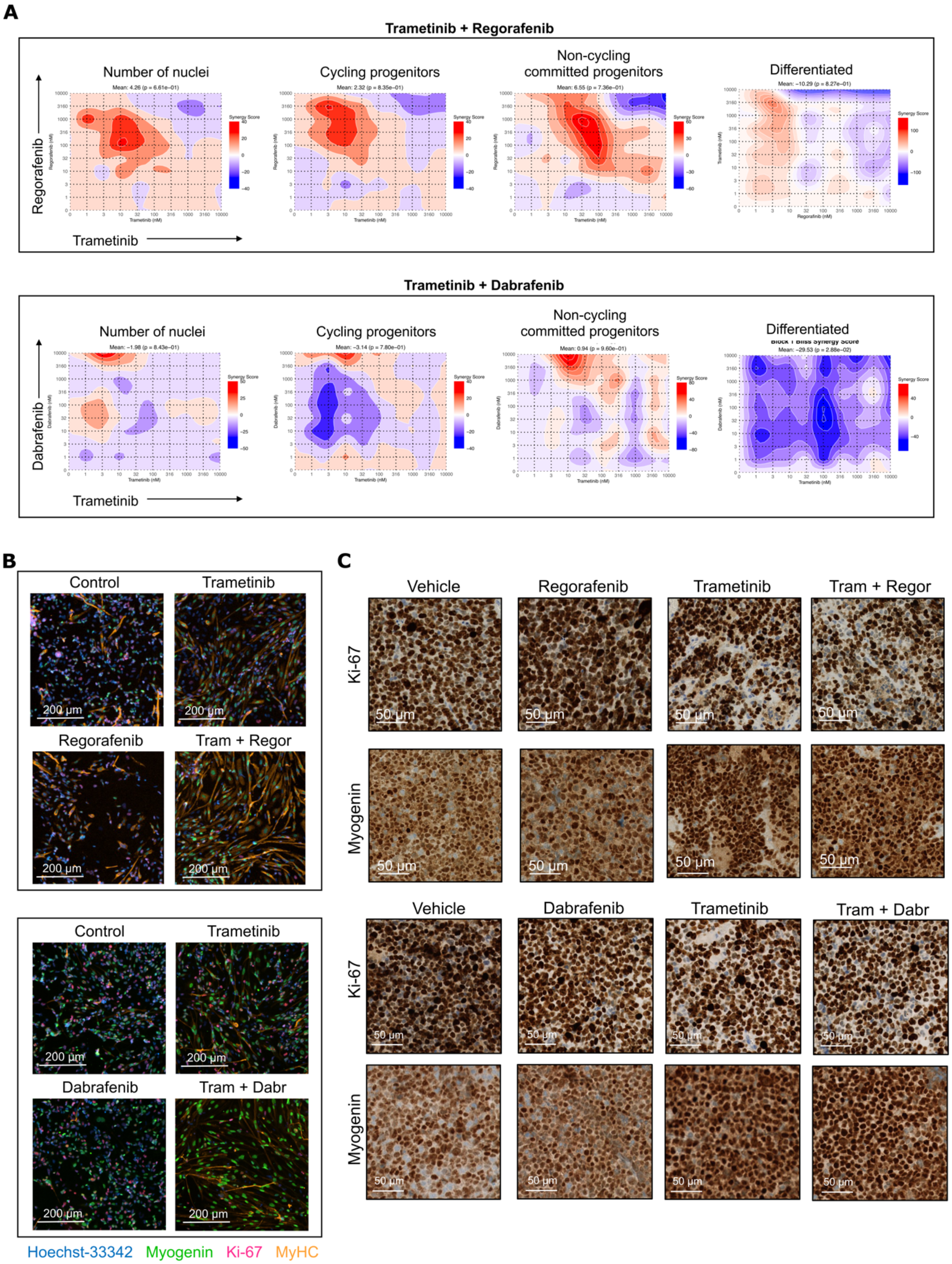

Fig. S7: Effect of the combination of trametinib with the RAS inhibitors dabrafenib or

regorafenib in aRMS primary cultures and PDXs.

A. Synergy maps of trametinib-regorafenib (top row) or trametinib-dabrafenib (bottom row) combinations. Cells from aRMS-1 (IC-pPDX-104) were exposed to the drugs for 72 hrs and then processed for MYOscopy. Synergy was calculated according to the Bliss model (*66*) on SynergyFinder (*67*). **B.** Representative images of immunofluorescence analysis of aRMS-1 (IC-pPDX-104) cells exposed to vehicle controls, 10 nM trametinib, 10 μM dabrafenib, 1 μM regorafenib, or to the corresponding combinations for 72 hrs. **C.** Expression of Ki-67 (proliferation marker) and myogenin (progenitor marker) as determined by immunohistochemistry in aRMS-1 (IC-pPDX-104) PDX tumors following *in vivo* treatment with 5 mg/kg trametinib, 15 mg/kg regorafenib or with their combination (top row), or with 1 mg/kg trametinib, 15 mg/kg dabrafenib or with their combination (bottom row).

Table S1. PDX characteristics and information on the clinical status of the patients.
Table S2. Table S2. Cluster markers of the integrated RMS scRNAseq dataset / Cluster markers of the integrated aRMS primary culture scRNAseq dataset / Cluster markers of the integrated eRMS primary culture scRNAseq dataset.
Table S3. Markers identified in previously published scRNAseq datasets of skeletal muscles (De Micheli et al., Cell Rep., 2020) / Markers identified in previously published scRNAseq datasets of skeletal muscles (Oprescu et al., iScience, 2020).
Table S4. Genes differentially expressed in Rh4 shPAX3::FOXO1 cells following treatment with doxycycline / Genes differentially expressed in KFR shPAX3::FOXO1 cells following treatment with doxycycline.
Table S5. Cellular composition of aRMS-1 (IC-pPDX-104) cells measured with MYOscopy following drug treatment / Cellular composition of aRMS-3 (IC-pPDX-35) cells measured with MYOscopy following drug treatment.
Table S6. Cellular composition of aRMS-1 (IC-pPDX-104) cells measured with MYOscopy following combinatorial drug treatment with a backbone of 50 nM trametinib.

Table S7. Tumor growth of PDX mice injected with aRMS-1 (IC-pPDX-104) cells subcutaneously and treated with trametinib-regorafenib / qRT-PCR of myogenic markers from aRMS-1 (IC-pPDX-104) tumors treated with trametinib+regorafenib / qRT-PCR of myogenic markers from aRMS-1 (IC-pPDX-104) tumors treated with trametinib+dabrafenib.

Table S8. Drug doses used for *in vivo* experiment in relation to the maximum tolerated doses in humans.

Table S9. PDX information / Hashtag antibody sequences used for multiplexing.

Table S10. Integration of aRMS scRNAseq and snRNAseq datasets / Integration of eRMS scRNAseq and snRNAseq datasets.

REFERENCES AND NOTES

1. T. H. H. Coorens, S. Behjati, Tracing and targeting the origins of childhood cancer. *Annu. Rev. Cancer Biol.* **6**, 35–47 (2022).

2. M. Filbin, M. Monje, Developmental origins and emerging therapeutic opportunities for childhood cancer. *Nat. Med.* **25**, 367–376 (2019).

3. R. L. Siegel, K. D. Miller, A. Jemal, Cancer statistics, 2020. *CA Cancer J. Clin.* **70**, 7–30 (2020).

4. D. Hanahan, Hallmarks of cancer: New dimensions. *Cancer Discov.* **12**, 31–46 (2022).

5. S. Jessa, A. Blanchet-Cohen, B. Krug, M. Vladoiu, M. Coutelier, D. Faury, B. Poreau, N. De Jay, S. Hébert, J. Monlong, W Todd Farmer, L. K. Donovan, Y. Hu, M. K. McConechy, F. M. G. Cavalli, L. G. Mikael, B. Ellezam, M. Richer, A. Allaire, A. G. Weil, J. Atkinson, J.-P. Farmer, R. W. R. Dudley, V. Larouche, L. Crevier, S. Albrecht, M. G. Filbin, H. Sartelet, P.-E. Lutz, C. Nagy, G. Turecki, S. Costantino, P. B. Dirks, K. K. Murai, G. Bourque, J. Ragoussis, L. Garzia, M. D. Taylor, N. Jabado, C. L. Kleinman, Stalled developmental programs at the root of pediatric brain tumors. *Nat. Genet.* **51**, 1702–1713 (2019).

6. J. Gojo, B. Englinger, L. Jiang, J. M. Hübner, M. K. L. Shaw, O. A. Hack, S. Madlener, D. Kirchhofer, I. Liu, J. Pyrdol, V. Hovestadt, E. Mazzola, N. D. Mathewson, M. Trissal, D. Lötsch, C. Dorfer, C. Haberler, A. Halfmann, L. Mayr, A. Peyrl, R. Geyeregger, B. Schwalm, M. Mauermann, K. W. Pajtler, T. Milde, M. E. Shore, J. E. Geduldig, K. Pelton, T. Czech, O. Ashenberg, K. W. Wucherpfennig, O. Rozenblatt-Rosen, S. Alexandrescu, K. L. Ligon, S. M. Pfister, A. Regev, I. Slavc, W. Berger, M. L. Suvà, M. Kool, M. G. Filbin, Single-cell RNA-seq reveals cellular hierarchies and impaired developmental trajectories in pediatric ependymoma. *Cancer Cell* **38**, 44–59.e9 (2020).

7. M. G. Filbin, I. Tirosh, V. Hovestadt, M. L. Shaw, L. E. Escalante, N. D. Mathewson, C. Neftel, N. Frank, K. Pelton, C. M. Hebert, C. Haberler, K. Yizhak, J. Gojo, K. Egervari, C. Mount, P. van Galen, D. M. Bonal, Q.-D. Nguyen, A. Beck, C. Sinai, T. Czech, C. Dorfer, L. Goumnerova, C. Lavarino, A. M. Carcaboso, J. Mora, R. Mylvaganam, C. C. Luo, A. Peyrl, M. Popović, A. Azizi, T. T. Batchelor, M. P. Frosch, M. Martinez-Lage, M. W. Kieran, P. Bandopadhayay, R.

Beroukhim, G. Fritsch, G. Getz, O. Rozenblatt-Rosen, K. W. Wucherpfennig, D. N. Louis, M. Monje, I. Slavc, K. L. Ligon, T. R. Golub, A. Regev, B. E. Bernstein, M. L. Suvà, Developmental and oncogenic programs in H3K27M gliomas dissected by single-cell RNA-seq. *Science* **360**, 331–335 (2018).

8. R. Dong, R. Yang, Y. Zhan, H. D. Lai, C. J. Ye, X. Y. Yao, W. Q. Luo, X. M. Cheng, J. J. Miao, J. F. Wang, B. H. Liu, X. Q. Liu, L. L. Xie, Y. Li, M. Zhang, L. Chen, W. C. Song, W. Qian, W. Q. Gao, Y. H. Tang, C. Y. Shen, W. Jiang, G. Chen, W. Yao, K. R. Dong, X. M. Xiao, S. Zheng, K. Li, J. Wang, Single-cell characterization of malignant phenotypes and developmental trajectories of adrenal neuroblastoma. *Cancer Cell* **38**, 716–733.e6 (2020).

9. S. X. Skapek, A. Ferrari, A. A. Gupta, P. J. Lupo, E. Butler, J. Shipley, F. G. Barr, D. S. Hawkins, Rhabdomyosarcoma. *Nat. Rev. Dis. Primers* **5**, 1 (2019).

10. S. Hettmer, A. J. Wagers, Muscling in: Uncovering the origins of rhabdomyosarcoma. *Nat. Med.* **16**, 171–173 (2010).

11. S. Kumar, E. Perlman, C. A. Harris, M. Raffeld, M. Tsokos, Myogenin is a specific marker for rhabdomyosarcoma: An immunohistochemical study in paraffin-embedded tissues. *Mod. Pathol.* **13**, 988–993 (2000).

12. N. J. Sebire, M. Malone, Myogenin and MyoD1 expression in paediatric rhabdomyosarcomas. *J. Clin. Pathol.* **56**, 412–416 (2003).

13. E. Charytonowicz, C. Cordon-Cardo, I. Matushansky, M. Ziman, Alveolar rhabdomyosarcoma: Is the cell of origin a mesenchymal stem cell? *Cancer Lett.* **279**, 126–136 (2009).

14. D. M. Langenau, M. D. Keefe, N. Y. Storer, J. R. Guyon, J. L. Kutok, X. le, W. Goessling, D. S. Neuberg, L. M. Kunkel, L. I. Zon, Effects of RAS on the genesis of embryonal rhabdomyosarcoma. *Genes Dev.* **21**, 1382–1395 (2007).

15. C. Keller, M. R. Capecchi, New genetic tactics to model alveolar rhabdomyosarcoma in the mouse. *Cancer Res.* **65**, 7530–7532 (2005).

16. C. J. Drummond, J. A. Hanna, M. R. Garcia, D. J. Devine, A. J. Heyrana, D. Finkelstein, J. E. Rehg, M. E. Hatley, Hedgehog pathway drives fusion-negative rhabdomyosarcoma initiated from non-myogenic endothelial progenitors. *Cancer Cell* **33**, 108–124.e5 (2018).

17. M. E. Hatley, W. Tang, M. R. Garcia, D. Finkelstein, D. P. Millay, N. Liu, J. Graff, R. L. Galindo, E. N. Olson, A mouse model of rhabdomyosarcoma originating from the adipocyte lineage. *Cancer Cell* **22**, 536–546 (2012).

18. A. S. Pappo, J. R. Anderson, W. M. Crist, M. D. Wharam, P. P. Breitfeld, D. Hawkins, R. B. Raney, R. B. Womer, D. M. Parham, S. J. Qualman, H. E. Grier, Survival after relapse in children and adolescents with rhabdomyosarcoma: A report from the intergroup rhabdomyosarcoma study group. *J. Clin. Oncol.* **17**, 3487–3493 (1999).

19. C. M. Heske, L. Mascarenhas, Relapsed rhabdomyosarcoma. *J. Clin. Med.* **10**, 804 (2021).

20. J. F. Shern, J. Selfe, E. Izquierdo, R. Patidar, H. C. Chou, Y. K. Song, M. E. Yohe, S. Sindiri, J. Wei, X. Wen, E. R. Rudzinski, D. A. Barkauskas, T. Lo, D. Hall, C. M. Linardic, D. Hughes, S. Jamal, M. Jenney, J. Chisholm, R. Brown, K. Jones, B. Hicks, P. Angelini, S. George, L. Chesler, M. Hubank, A. Kelsey, S. A. Gatz, S. X. Skapek, D. S. Hawkins, J. M. Shipley, J. Khan, Genomic classification and clinical outcome in rhabdomyosarcoma: A report from an international consortium. *J. Clin. Oncol.* **39**, 2859–2871 (2021).

21. J. F. Shern, L. Chen, J. Chmielecki, J. S. Wei, R. Patidar, M. Rosenberg, L. Ambrogio, D. Auclair, J. Wang, Y. K. Song, C. Tolman, L. Hurd, H. Liao, S. Zhang, D. Bogen, A. S. Brohl, S. Sindiri, D. Catchpoole, T. Badgett, G. Getz, J. Mora, J. R. Anderson, S. X. Skapek, F. G. Barr, M. Meyerson, D. S. Hawkins, J. Khan, Comprehensive genomic analysis of rhabdomyosarcoma reveals a landscape of alterations affecting a common genetic axis in fusion-positive and fusion-negative tumors. *Cancer Discov.* **4**, 216–231 (2014).

22. M. Wachtel, B. W. Schäfer, PAX3-FOXO1: Zooming in on an “undruggable” target. *Semin. Cancer Biol.* **50**, 115–123 (2018).

23. F. Dela Cruz, I. Matushansky, Solid tumor differentiation therapy—Is it possible? *Oncotarget* **3**, 559–567 (2012).

24. H. De Thé, Differentiation therapy revisited. *Nat. Rev. Cancer* **18**, 117–127 (2018).

25. N. Garcia, V. del-Pozo, M. Yohe, C. Goodwin, T. Shackleford, L. Wang, K. Baxi, Y. Chen, M. Ignatius, K. Wood, P. Houghton, A. Vaseva, Abstract LB251: Vertical inhibition of the RAF MEK ERK cascade induces myogenic differentiation, apoptosis and tumor regression in H/NRAS Q61X mutant rhabdomyosarcoma. *Mol. Cancer Ther.* **81**, LB251 (2021).

26. M. E. Yohe, B. E. Gryder, J. F. Shern, Y. K. Song, H.-C. Chou, S. Sindiri, A. Mendoza, R. Patidar, X. Zhang, R. Guha, D. Butcher, K. A. Isanogle, C. M. Robinson, X. Luo, J.-Q. Chen, A. Walton, P. Awasthi, E. F. Edmondson, S. Difilippantonio, J. S. Wei, K. Zhao, M. Ferrer, C. J. Thomas, J. Khan, MEK inhibition induces MYOG and remodels super-enhancers in RAS-driven rhabdomyosarcoma. *Sci. Transl. Med.* **10**, eaan4470 (2018).

27. S. Pomella, P. Sreenivas, B. E. Gryder, L. Wang, D. Milewski, M. Cassandri, K. Baxi, N. R. Hensch, E. Carcarino, Y. Song, H.-C. Chou, M. E. Yohe, B. Z. Stanton, B. Amadio, I. Caruana, C. De Stefanis, R. De Vito, F. Locatelli, Y. Chen, E. Y. Chen, P. Houghton, J. Khan, R. Rota, M. S. Ignatius, Interaction between SNAI2 and MYOD enhances oncogenesis and suppresses differentiation in fusion negative rhabdomyosarcoma. *Nat. Commun.* **12**, 192 (2021).

28. E. J. Fox, L. A. Loeb, Cancer: One cell at a time. *Nature* **512**, 143–144 (2014).

29. T. Stuart, A. Butler, P. Hoffman, C. Hafemeister, E. Papalexi, W. M. Mauck III, Y. Hao, M. Stoeckius, P. Smibert, R. Satija, Comprehensive integration of single-cell data. *Cell* **177**, 1888–1902.e21 (2019).

30. Y. Zhou, B. Zhou, L. Pache, M. Chang, A. H. Khodabakhshi, O. Tanaseichuk, C. Benner, S. K. Chanda, Metascape provides a biologist-oriented resource for the analysis of systems-level datasets. *Nat. Commun.* **10**, 1523 (2019).

31. S. N. Oprescu, F. Yue, J. Qiu, L. F. Brito, S. Kuang, Temporal dynamics and heterogeneity of cell populations during skeletal muscle regeneration. *iScience* **23**, 100993 (2020).

32. A. J. De Micheli, E. J. Laurilliard, C. L. Heinke, H. Ravichandran, P. Fraczek, S. Soueid-Baumgarten, I. De Vlaminck, O. Elemento, B. D. Cosgrove, Single-cell analysis of the muscle stem cell hierarchy identifies heterotypic communication signals involved in skeletal muscle regeneration. *Cell Rep.* **30**, 3583–3595.e5 (2020).

33. E. Z. Macosko, A. Basu, R. Satija, J. Nemesh, K. Shekhar, M. Goldman, I. Tirosh, A. R. Bialas, N. Kamitaki, E. M. Martersteck, J. J. Trombetta, D. A. Weitz, J. R. Sanes, A. K. Shalek, A. Regev, S. A. McCarroll, Highly parallel genome-wide expression profiling of individual cells using nanoliter droplets. *Cell* **161**, 1202–1214 (2015).

34. I. Tirosh, B. Izar, S. M. Prakadan, M. H. Wadsworth II, D. Treacy, J. J. Trombetta, A. Rotem, C. Rodman, C. Lian, G. Murphy, M. Fallahi-Sichani, K. Dutton-Regester, J. R. Lin, O. Cohen, P. Shah, D. Lu, A. S. Genshaft, T. K. Hughes, C. G. K. Ziegler, S. W. Kazer, A. Gaillard, K. E. Kolb, A. C. Villani, C. M. Johannessen, A. Y. Andreev, E. M. van Allen, M. Bertagnolli, P. K. Sorger, R. J. Sullivan, K. T. Flaherty, D. T. Frederick, J. Jané-Valbuena, C. H. Yoon, O. Rozenblatt-Rosen, A. K. Shalek, A. Regev, L. A. Garraway, Dissecting the multicellular ecosystem of metastatic melanoma by single-cell RNA-seq. *Science* **352**, 189–196 (2016).

35. E. Davicioni, F. Graf Finckenstein, V. Shahbazian, J. D. Buckley, T. J. Triche, M. J. Anderson, Identification of a PAX-FKHR gene expression signature that defines molecular classes and determines the prognosis of alveolar rhabdomyosarcomas. *Cancer Res.* **66**, 6936–6946 (2006).

36. A. G. Patel, X. Chen, X. Huang, M. R. Clay, N. Komorova, M. J. Krasin, A. Pappo, H. Tillman, B. A. Orr, J. M. Evoy, B. Gordon, K. Blankenship, C. Reilly, X. Zhou, J. L. Norrie, A. Karlstrom, J. Yu, D. Wodarz, E. Stewart, M. A. Dyer, The myogenesis program drives clonal selection and drug resistance in rhabdomyosarcoma. *Dev. Cell* **57**, 1226–1240.e8 (2022).

37. E. Porpiglia, N. Samusik, A. T. V. Ho, B. D. Cosgrove, T. Mai, K. L. Davis, A. Jager, G. P. Nolan, S. C. Bendall, W. J. Fantl, H. M. Blau, High-resolution myogenic lineage mapping by single-cell mass cytometry. *Nat. Cell Biol.* **19**, 558–567 (2017).

38. N. Samusik, Z. Good, M. H. Spitzer, K. L. Davis, G. P. Nolan, Automated mapping of phenotype space with single-cell data. *Nat. Methods* **13**, 493–496 (2016).

39. G. K. Behbehani, S. C. Bendall, M. R. Clutter, W. J. Fantl, G. P. Nolan, Single-cell mass cytometry adapted to measurements of the cell cycle. *Cytometry A* **81**, 552–566 (2012).

40. K. Street, D. Risso, R. B. Fletcher, D. das, J. Ngai, N. Yosef, E. Purdom, S. Dudoit, Slingshot: Cell lineage and pseudotime inference for single-cell transcriptomics. *BMC Genomics* **19**, 477 (2018).

41. K. R. Moon, D. van Dijk, Z. Wang, S. Gigante, D. B. Burkhardt, W. S. Chen, K. Yim, A. van den Elzen, M. J. Hirn, R. R. Coifman, N. B. Ivanova, G. Wolf, S. Krishnaswamy, Visualizing structure and transitions in high-dimensional biological data. *Nat. Biotechnol.* **37**, 1482–1492 (2019).

42. H. Xi, J. Langerman, S. Sabri, P. Chien, C. S. Young, S. Younesi, M. Hicks, K. Gonzalez, W. Fujiwara, J. Marzi, S. Liebscher, M. Spencer, B. Van Handel, D. Evseenko, K. Schenke-Layland, K. Plath, A. D. Pyle, A human skeletal muscle atlas identifies the trajectories of stem and progenitor cells across development and from human pluripotent stem cells. *Cell Stem Cell* **27**, 158–176.e10 (2020).

43. J. Ommer, J. L. Selfe, M. Wachtel, E. M. O'Brien, D. Laubscher, M. Roemmele, S. Kasper, O. Delattre, D. Surdez, G. Petts, A. Kelsey, J. Shipley, B. W. Schäfer, Aurora a kinase inhibition destabilizes PAX3-FOXO1 and MYCN and synergizes with navitoclax to induce rhabdomyosarcoma cell death. *Cancer Res.* **80**, 832–842 (2020).

44. S. M. Wilhelm, J. Dumas, L. Adnane, M. Lynch, C. A. Carter, G. Schütz, K. H. Thierauch, D. Zopf, Regorafenib (BAY 73-4506): A new oral multikinase inhibitor of angiogenic, stromal and oncogenic receptor tyrosine kinases with potent preclinical antitumor activity. *Int. J. Cancer* **129**, 245–255 (2011).

45. L. Liu, Y. Cao, C. Chen, X. Zhang, A. McNabola, D. Wilkie, S. Wilhelm, M. Lynch, C. Carter, Sorafenib blocks the RAF/MEK/ERK pathway, inhibits tumor angiogenesis, and induces tumor cell apoptosis in hepatocellular carcinoma model PLC/PRF/5. *Cancer Res.* **66**, 11851–11858 (2006).

46. T. R. Rheault, J. C. Stellwagen, G. M. Adjabeng, K. R. Hornberger, K. G. Petrov, A. G. Waterson, S. H. Dickerson, R. A. Mook Jr, S. G. Laquerre, A. J. King, O. W. Rossanese, M. R. Arnone, K. N. Smitheman, L. S. Kane-Carson, C. Han, G. S. Moorthy, K. G. Moss, D. E. Uehling, Discovery of dabrafenib: A selective inhibitor of Raf kinases with antitumor activity against B-Raf-driven tumors. *ACS Med. Chem. Lett.* **4**, 358–362 (2013).

47. S. Knispel, L. Zimmer, T. Kanaki, S. Ugurel, D. Schadendorf, E. Livingstone, The safety and efficacy of dabrafenib and trametinib for the treatment of melanoma. *Expert Opin. Drug Saf.* **17**, 73–87 (2018).

48. R. J. Kelly, Dabrafenib and trametinib for the treatment of non–small cell lung cancer. *Expert Rev. Anticancer Ther.* **18**, 1063–1068 (2018).

49. Y. Wei, Q. Qin, C. Yan, M. N. Hayes, S. P. Garcia, H. Xi, D. Do, A. H. Jin, T. C. Eng, K. M. McCarthy, A. Adhikari, M. L. Onozato, D. Spentzos, G. P. Neilsen, A. John Iafrate, L. H. Wexler, A. D. Pyle, M. L. Suvà, F. D. Cruz, L. Pinello, D. M. Langenau, Single-cell analysis and functional characterization uncover the stem cell hierarchies and developmental origins of rhabdomyosarcoma. *Nat. Cancer* **3**, 961–975 (2022).

50. C. P. Couturier, S. Ayyadhury, P. U. le, J. Nadaf, J. Monlong, G. Riva, R. Allache, S. Baig, X. Yan, M. Bourgey, C. Lee, Y. C. D. Wang, V. Wee Yong, M. C. Guiot, H. Najafabadi, B. Misic, J. Antel, G. Bourque, J. Ragoussis, K. Petrecca, Single-cell RNA-seq reveals that glioblastoma recapitulates a normal neurodevelopmental hierarchy. *Nat. Commun.* **11** (2020).

51. L. Zhang, X. He, X. Liu, F. Zhang, L Frank Huang, A. S. Potter, L. Xu, W. Zhou, T. Zheng, Z. Luo, K. P. Berry, A. Pribnow, S. M. Smith, C. Fuller, B. V. Jones, M. Fouladi, R. Drissi, Z.-J. Yang, W Clay Gustafson, M. Remke, S. L. Pomeroy, E. J. Girard, J. M. Olson, A. S. Morrissy, M. C. Vladoiu, J. Zhang, W. Tian, M. Xin, M. D. Taylor, S Steven Potter, M. F. Roussel, W. A. Weiss, Q. R. Lu, Single-cell transcriptomics in medulloblastoma reveals tumor-initiating progenitors and oncogenic cascades during tumorigenesis and relapse. *Cancer Cell* **36**, 302–318.e7 (2019).

52. M. Generali, S. Satheesha, P. K. Bode, D. Wanner, B. W. Schäfer, E. A. Casanova, High frequency of tumor propagating cells in fusion-positive rhabdomyosarcoma. *Genes* **12** (2021).

53. M. Ebauer, M. Wachtel, F. K. Niggli, B. W. Schäfer, Comparative expression profiling identifies an in vivo target gene signature with TFAP2B as a mediator of the survival function of PAX3/FKHR. *Oncogene* **26**, 7267–7281 (2007).

54. S. Boudjadi, P. R. Pandey, B. Chatterjee, T. H. Nguyen, W. Sun, F. G. Barr, A fusion transcription factor⇓driven cancer progresses to a fusion-independent relapse via constitutive activation of a downstream transcriptional target. *Cancer Res.* **81**, 2930–2942 (2021).

55. S. M. Shaffer, M. C. Dunagin, S. R. Torborg, E. A. Torre, B. Emert, C. Krepler, M. Beqiri, K. Sproesser, P. A. Brafford, M. Xiao, E. Eggan, I. N. Anastopoulos, C. A. Vargas-Garcia, A. Singh, K. L. Nathanson, M. Herlyn, A. Raj, Rare cell variability and drug-induced reprogramming as a mode of cancer drug resistance. *Nature* **546**, 431–435 (2017).

56. S. V. Sharma, D. Y. Lee, B. Li, M. P. Quinlan, F. Takahashi, S. Maheswaran, U. McDermott, N. Azizian, L. Zou, M. A. Fischbach, K. K. Wong, K. Brandstetter, B. Wittner, S. Ramaswamy, M. Classon, J. Settleman, A chromatin-mediated reversible drug-tolerant state in cancer cell subpopulations. *Cell* **141**, 69–80 (2010).

57. J. M. Rosen, C. T. Jordan, The increasing complexity of the cancer stem cell paradigm. *Science* **324**, 1670–1673 (2009).

58. P. B. Gupta, I. Pastushenko, A. Skibinski, C. Blanpain, C. Kuperwasser, Phenotypic plasticity: Driver of cancer initiation, progression, and therapy resistance. *Cell Stem Cell* **24**, 65–78 (2019).

59. G. Manzella, L. D. Schreck, W. B. Breunis, J. Molenaar, H. Merks, F. G. Barr, W. Sun, M. Römmele, L. Zhang, J. Tchinda, Q. A. Ngo, P. Bode, O. Delattre, D. Surdez, B. Rekhi, F. K. Niggli, B. W. Schäfer, M. Wachtel, Phenotypic profiling with a living biobank of primary rhabdomyosarcoma unravels disease heterogeneity and AKT sensitivity. *Nat. Commun.* **11**, 4629 (2020).

60. D. Surdez, L. Landuzzi, K. Scotlandi, M. C. Manara, Ewing sarcoma PDX models. *Methods Mol. Biol.* **2226**, 223–242 (2021).

61. A. J. Garvin, W. S. Stanley, J. L. Sullivan, D. D. Sens, The in vitro growth, heterotransplantation, and differentiation of a human rhabdomyosarcoma cell line. *Am. J. Pathol.* **125**, 208–217 (1986).

62. M. Stoeckius, S. Zheng, B. Houck-Loomis, S. Hao, B. Z. Yeung, W. M. Mauck III, P. Smibert, R. Satija, Cell hashing with barcoded antibodies enables multiplexing and doublet detection for single cell genomics. *Genome Biol.* **19**, 224 (2018).

63. S. Durinck, P. T. Spellman, E. Birney, W. Huber, Mapping identifiers for the integration of genomic datasets with the R/Bioconductor package biomaRt. *Nat. Protoc.* **4**, 1184–1191 (2009).

64. H. G. Fienberg, E. F. Simonds, W. J. Fantl, G. P. Nolan, B. Bodenmiller, A platinum-based covalent viability reagent for single-cell mass cytometry. *Cytometry A* **81**, 467–475 (2012).

65. R. Finck, E. F. Simonds, A. Jager, S. Krishnaswamy, K. Sachs, W. Fantl, D. Pe'er, G. P. Nolan, S. C. Bendall, Normalization of mass cytometry data with bead standards. *Cytometry A* **83**, 483–494 (2013).

66. A. McDavid, G. Finak, P. K. Chattopadyay, M. Dominguez, L. Lamoreaux, S. S. Ma, M. Roederer, R. Gottardo, Data exploration, quality control and testing in single-cell qPCR-based gene expression experiments. *Bioinformatics* **29**, 461–467 (2013).

67. C. I. Bliss, The toxicity of poisons applied JOINTLY1. *Ann. Appl. Biol.* **26**, 585–615 (1939).

68. S. Zheng, W. Wang, J. Aldahdooh, A. Malyutina, T. Shadbahr, Z. Tanoli, A. Pessia, J. Tang, SynergyFinder Plus: Toward better interpretation and annotation of drug combination screening datasets. *Genomics Proteomics Bioinformatics* **20**, 587–596 (2022).
